# Cobalt-catalysed alkene hydrogenation: a metallacycle can explain the hydroxyl activating effect and the diastereoselectivity†
†Electronic supplementary information (ESI) available: Computational and experimental details, calculated reaction profiles, optimised coordinates, NMR and X-ray data. See DOI: 10.1039/c8sc01315b


**DOI:** 10.1039/c8sc01315b

**Published:** 2018-05-04

**Authors:** Glenn R. Morello, Hongyu Zhong, Paul J. Chirik, Kathrin H. Hopmann

**Affiliations:** a Hylleraas Centre for Quantum Molecular Sciences , Department of Chemistry , University of Tromsø – The Arctic University of Norway , N-9037 Tromsø , Norway . Email: kathrin.hopmann@uit.no; b Department of Chemistry , Princeton University , New Jersey 08544 , USA

## Abstract

Bis(phosphine)cobalt dialkyl complexes have been reported to be highly active in the hydrogenation of tri-substituted alkenes bearing hydroxyl substituents. Alkene substrates containing ether, ester, or ketone substituents show minimal reactivity, indicating an activating effect of the hydroxyl group. The mechanistic details of bis(phosphine)cobalt-catalysed hydrogenation were recently evaluated computationally (X. Ma, M. Lei, *J. Org. Chem.* 2017, **82**, 2703–2712) and a Co(0)–Co(ii) redox mechanism was proposed. However, the activating effect of the hydroxyl substituent and the accompanying high diastereoselectivity were not studied. Here we report a computational study rationalizing the role of the hydroxyl group through a key metallacycle species. The metallacycle is part of a non-redox catalytic pathway proceeding through Co(ii) intermediates throughout. The preference for alcohol over ether substrates and the high diastereoselectivity of terpinen-4-ol hydrogenation are correctly predicted in computations adopting the new pathway, whereas the alternative redox mechanism predicts ethers rather than alcohols to be more reactive substrates. Additional experimental evidence supports the role of the hydroxyl group in the metallacycle mechanism. Our work highlights the importance of employing known substrate preferences and stereoselectivities to test the validity of computationally proposed reaction pathways.

## Abstract 2

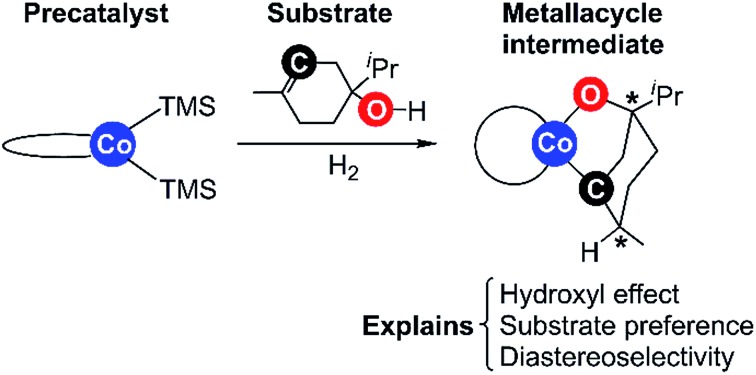
A new non-redox metallacycle mechanism explains the substrate preference, the diastereoselectivity, and the hydroxyl activating effect in cobalt-catalyzed alkene hydrogenation.

## Introduction

The majority of homogeneous hydrogenation catalysts employed today are based on precious metals such as rhodium, ruthenium, or iridium. During the last two decades, there has been an increasing focus on developing hydrogenation catalysts involving more earth abundant transition metals. Hydrogenation of alkenes or carbonyl compounds with transition metal complexes based on iron, cobalt, nickel, or manganese has been achieved by several groups, including those of Budzelaar,^1^ Chirik,^2^ Casey,^3^ Milstein,^4^ Hanson,^5^ Peters,^6^ Kempe,^7^ Morris,^8^ Fout,^9^ and Beller.^10^ Despite these promising results, more efforts are required to develop non-precious catalysts for diastereo- and enantioselective hydrogenation reactions. To date only a limited number of non-precious metal complexes are able to catalyse enantioselective alkene hydrogenations.2d,^11^,^12^,^13^

**Fig. 1:**
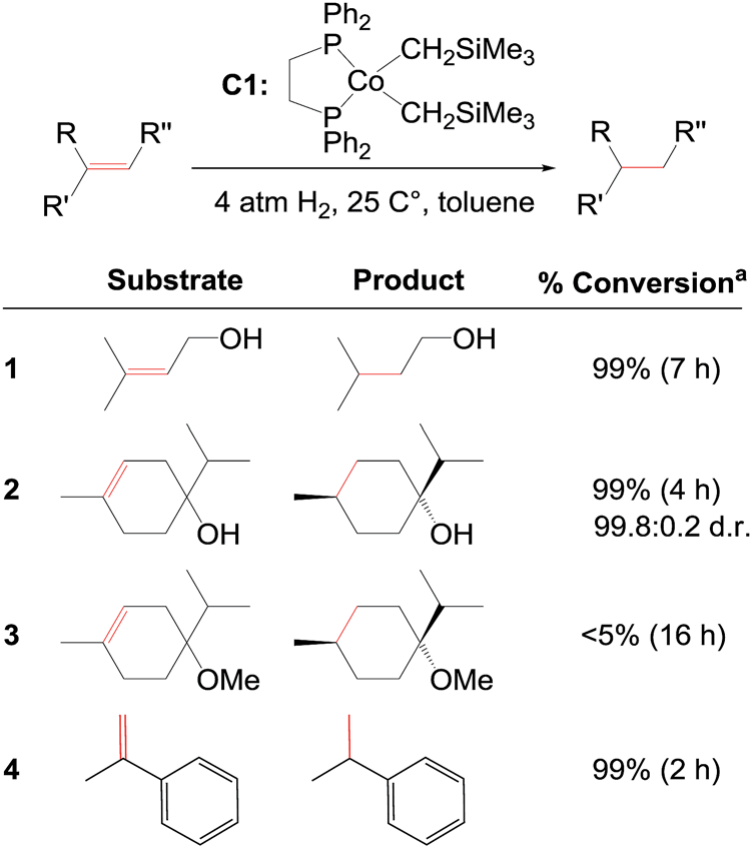
**C1**-catalysed alkene hydrogenation (data from ref. 2f). ^a^5% **C1** for entry 1 and 3, 1% **C1** for entry 2 and 4.

Recently, Chirik and co-workers reported bis(phosphine)cobalt dialkyl complexes for the hydrogenation of alkenes under mild conditions (Fig. 1).2*f* A significant activating effect by hydroxyl groups was observed for the cobalt catalysts. Ether, ester, or ketone groups did not provide such an effect.2*f* The reported cobalt dialkyl catalyst dppeCo(CH_2_SiMe_3_)_2_ (**C1**, dppe = 1,2-bis(diphenylphosphino)ethane) catalyses hydrogenation of terpinen-4-ol providing 99% conversion (entry 2, Fig. 1), whereas the corresponding methyl ether displays <5% conversion despite higher catalyst loading and longer reaction time (entry 3, Fig. 1).2*f* Interestingly, hydrogenation of terpinen-4-ol gives a high diastereoselectivity with a diastereomeric ratio (d.r.) of 99.8 : 0.2 (entry 2, Fig. 1). Compared to tri-substituted alkenes, di-substituted terminal alkenes could be hydrogenated without a hydroxyl group present (entry 4, Fig. 1).

**Fig. 2:**
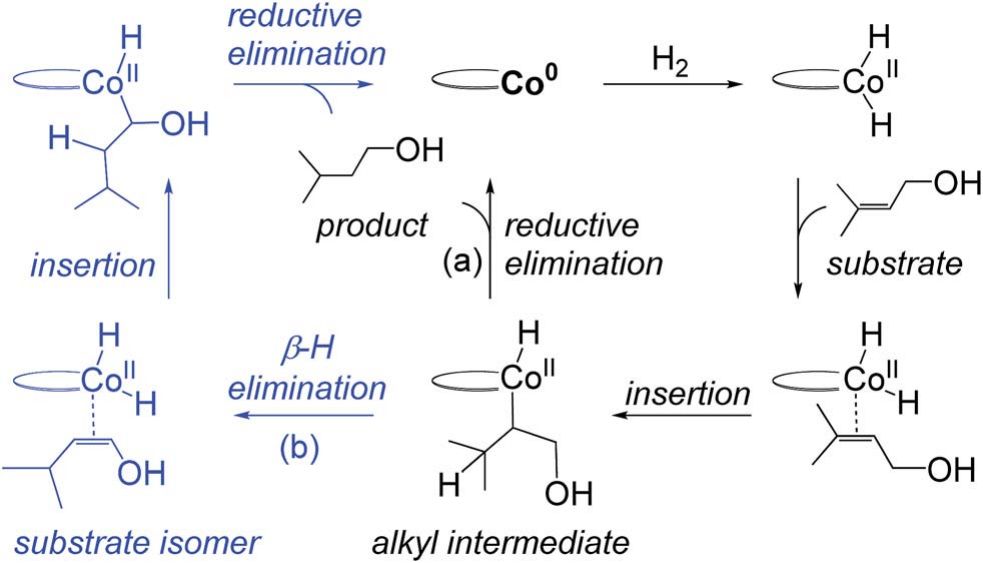
Previously proposed redox pathway for **C1** (based on results in ref. 14). A Co(0) species oxidatively adds H_2_ to form a Co(ii)–dihydride, followed by alkene insertion to form an alkyl intermediate. The alkyl undergoes reductive elimination (a) or β-H elimination (b) to yield the product or alkene isomers.

Recent computational work by Ma and Lei on **C1** suggests that hydrogenation of hydroxylated tri-substituted alkenes proceeds through a Co(0)–Co(ii) redox mechanism (Fig. 2).^14^ The proposed catalytic cycle starts with oxidative addition of H_2_ to a Co(0) species generating a Co(ii)–dihydride, followed by substrate insertion to give an alkyl intermediate. The inter-mediate may undergo direct reductive elimination to yield the product alkane and regenerate Co(0) (Fig. 2, path (a)) or may proceed *via* β-hydrogen elimination to form an alkene regioisomer, followed by substrate reinsertion and reductive elimination (Fig. 2, path (b)). Regardless if alkene isomerization occurs, the same product is formed and the overall pathway involves a cycling between Co(0) and Co(ii) oxidation states. The role of the hydroxyl group was not considered in the previous analysis.^14^

Here we present computational and experimental results, which provide novel insights into the mechanism of **C1**-catalysed directed hydrogenation. On basis of our results, we propose that hydrogenation of hydroxylated alkenes occurs through a non-redox reaction pathway proceeding through a metallacycle, which is formed through activation of the hydroxyl group. The metallacycle mechanism correctly predicts the catalyst's preference for hydroxylated alkenes and the high diastereoselectivity observed in hydrogenation of terpinen-4-ol.

## Results and discussion

### Computational results

#### Redox pathway

**Fig. 3:**
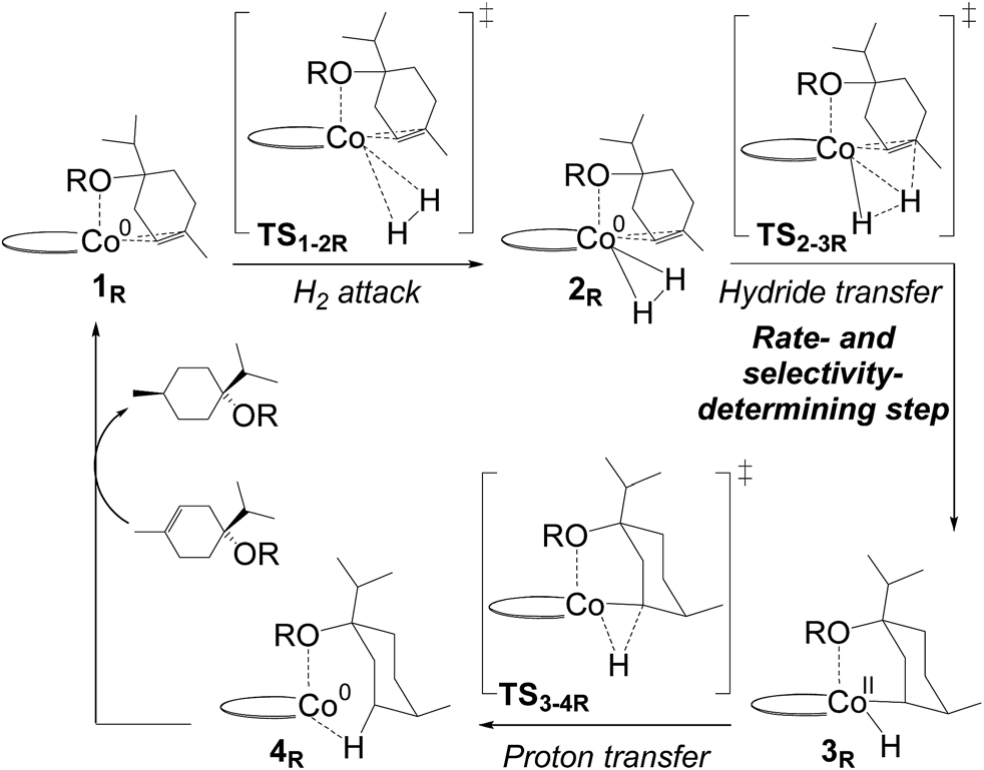
Co(0)–Co(ii) redox mechanism computed for **C1**-catalysed hydrogenation of terpinen-4-ol (R = H) and its methoxy-derivative (R = CH_3_). Computed energies are shown in Fig. 4. Terpinen-4-ol prefers an alternative mechanism, Fig. 5.

We performed a quantum chemical analysis of a full molecular model of **C1** with different substrates using B3LYP-D3 with the solvent model IEFPCM (for computational details see ESI†). Initially, we evaluated if **C1** employs a redox mechanism (Fig. 3), similar to the mechanism proposed by Ma and Lei (Fig. 2).^14^ Our redox mechanism differs slightly, as we find that substrate coordination precedes addition of H_2_, and we conclude that the intermediate formed upon H_2_ coordination prefers a Co(0)–H_2_ structure over a Co(ii)–dihydride configuration (the dihydride is 2.6 kcal mol^–1^ higher in energy, Fig. S1, ESI†). The overall hydrogenation steps of ours (Fig. 3) and the previous redox mechanism (Fig. 2) are otherwise identical. The rate-determining step is the formation of the alkyl-intermediate (**TS_2-3R_**, Fig. 3), involving transfer of a hydride to the methyl-substituted carbon (the pathway involving hydride transfer to the other carbon has a larger barrier, Fig. S2, ESI†). The alkyl-intermediate then undergoes reductive elimination to form the alkane product (a β-H elimination pathway was also evaluated, but is higher in energy, Fig. S3, ESI†).

**Fig. 4:**
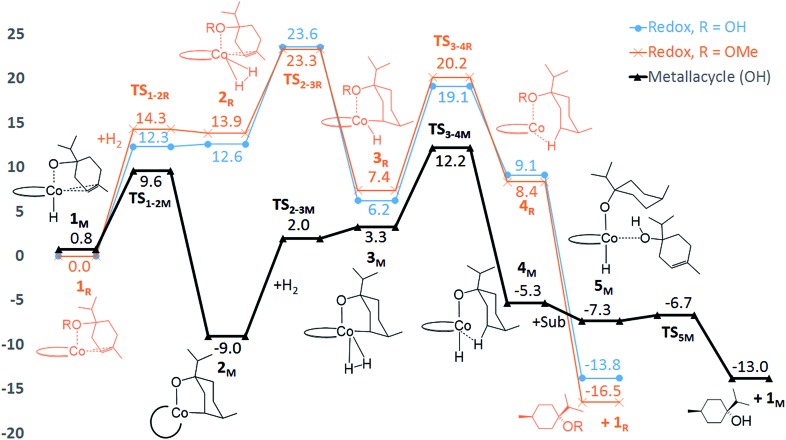
Computed free energies (kcal mol^–1^) for **C1**-catalysed hydrogenation. The redox mechanism is shown for substrates terpinen-4-ol (R = OH, blue line) and its methoxy derivative (R = OMe, orange line). The metallacycle mechanism is shown for terpinen-4-ol (black line). All energies are referenced to the **1_R_** state of each substrate.

In order to evaluate the validity of the redox mechanism, we considered the substrate preference with this pathway. As previously determined in experiments, terpinen-4-ol (Fig. 1, entry 2) is the preferred substrate compared to its methoxy derivative (Fig. 1, entry 3). With the redox mechanism, the overall barriers are calculated to be +23.6 kcal mol^–1^ for terpinen-4-ol and +23.3 kcal mol^–1^ for the methoxy-derivative, indicating a slight preference for the methoxy-substrate (Fig. 4). This is in disagreement with the experimentally observed strong preference for the alcohol substrate (Fig. 1). Analysis of the substrate selectivity indicates that **C1**-catalysed hydrogenation may not proceed through the previously proposed redox mechanism.

#### Metallacycle pathway

**Fig. 5:**
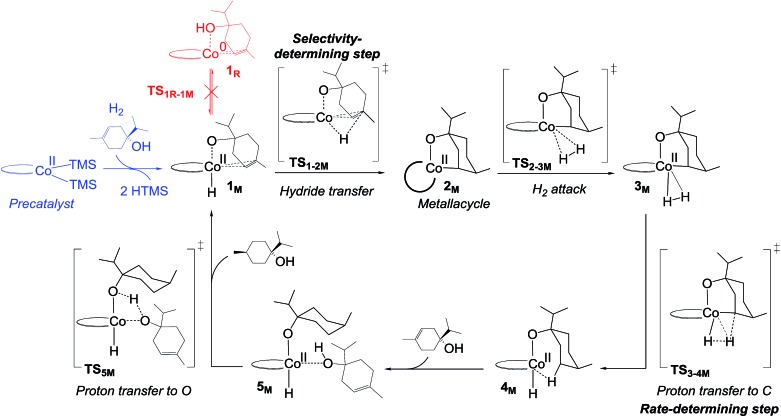
Non-redox metallacycle mechanism proposed for bis(phosphine)cobalt-catalysed hydrogenation of terpinen-4-ol. For details on the activation of the precatalyst, see Fig. S5, ESI.†

**Fig. 6:**
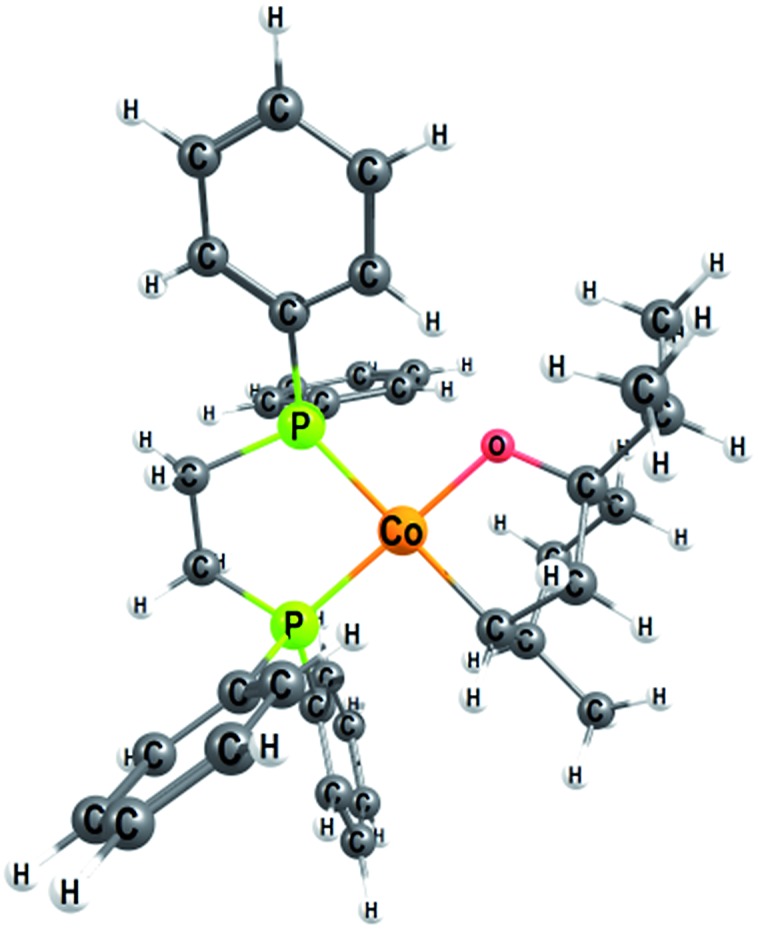
Optimised geometry of the metallacycle **2_M_**, a Co(ii) open shell species (*S* = 1/2) with a planar configuration around the cobalt atom.

We explored several alternative mechanistic possibilities (Fig. S2–S4, ESI†), of which only the preferred metallacycle pathway is discussed here (Fig. 5). We propose that reaction between the precatalyst **C1** and the substrate terpinen-4-ol under H_2_ results in generation of a cobalt(monohydride)(alkoxide) species, which we suggest constitutes the active catalyst **1_M_** (Fig. 5). Interestingly, formation of **1_M_** from the precatalyst **C1** is preferred over formation of the dihydride species implicated in the redox mechanism (see Fig. S5 ESI† for computed energies). **1_M_** can then undergo a hydride transfer to the double bond of the bound substrate to form the metallacycle **2_M_** (Fig. 5, for optimised geometry see Fig. 6), which is –9.8 kcal mol^–1^ below **1_M_** (Fig. 4). Subsequent coordination of H_2_ to the metallacycle appears facile, with a cost of 11 kcal mol^–1^ relative to **2_M_** (**TS_2-3M_**, Fig. 4). The barrier for H_2_ attack may be slightly underestimated, given that **3_M_** is ∼1 kcal mol^–1^ higher in energy than **TS_2-3M_**. Proton transfer from H_2_ to the alkyl carbon is the rate-limiting step (**TS_3-4M_**, 21.3 kcal mol^–1^ relative to **2_M_**, Fig. 4) and provides the hydrogenated alkane, which still exhibits a deprotonated hydroxyl coordinating to cobalt. In the final step, another substrate molecule transfers its hydroxyl proton to the alkane oxygen (**TS_5M_**), regenerating **1_M_** and turning over the cycle. The cobalt center has a formal oxidation state of +2 throughout (Fig. 5). Although a Co(0) species in principle can be formed in one step from the Co(ii) species **1_M_** through reductive elimination of the substrate (**TS_1R-1M_**, Fig. 5), this transformation has a prohibitively high barrier (36.5 kcal mol^–1^ relative to **2_M_**, Fig. S6, ESI†).

The calculated free energies show that the metallacycle **2_M_** is facile to form and that it is the lowest-lying intermediate computed here (Fig. 4), implying that other reaction pathways must be referenced to **2_M_**, even if the metallacycle constitutes an off-cycle species to these. The barrier for hydrogenation of terpinen-4-ol *via* the redox mechanism thus raises from 23.6 kcal mol^–1^ to 32.6 kcal mol^–1^ (**TS_2-3R_** relative to **2M**, Fig. 4). The barrier for the metallacycle mechanism is instead 21.3 kcal mol^–1^ (**TS_3-4M_** relative to **2M**, Fig. 4). Although DFT protocols may exhibit an error of some kcal mol^–1^,^15^ we consider a difference of 11.3 kcal mol^–1^ between the redox and the metallacycle mechanism to be more than significant to conclude that the metallacycle pathway is preferred for hydrogenation of terpinen-4-ol.

#### Analysis of the substrate preference

**Fig. 7:**
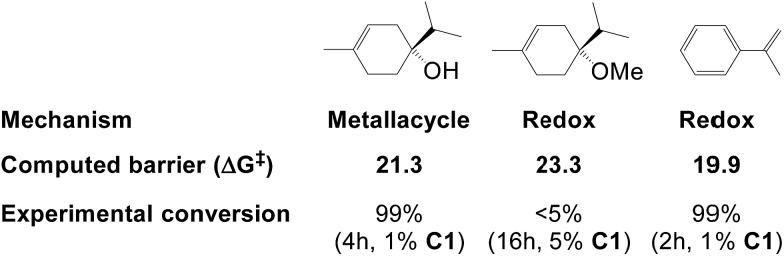
Hydrogenation barriers (kcal mol^–1^) computed here for three substrates and their experimental conversion (from ref. 2f).

The metallacycle mechanism (Fig. 5) is only accessible for substrates containing a hydroxyl group. For other substrates, an alternative hydrogenation mechanism must operate, which may be the redox pathway (Fig. 3). We have compared the computed barriers for three substrates (Fig. 7) to validate our mechanistic proposals.

Hydrogenation of terpinen-4-ol preferably occurs *via* the metallacycle pathway, with a computed barrier of 21.3 kcal mol^–1^. Redox hydrogenation of the methoxy-derivative of terpinen-4-ol has a computed barrier of 23.3 kcal mol^–1^, whereas the lowest computed barrier for redox hydrogenation of α-methylstyrene is 19.9 kcal mol^–1^ (four possible pathways were modelled, see ESI, Fig. S7†). These results are in excellent agreement with experiment (Fig. 1), which showed 99% conversion of terpinen-4-ol and α-methylstyrene (after 4 and 2 hours, respectively), whereas the methoxy substrate gave <5% conversion after 16 hours. The barrier difference of 2 kcal mol^–1^ between terpinen-4-ol (barrier 21.3 kcal mol^–1^) and its methoxy derivative (barrier 23.3 kcal mol^–1^) translates roughly to a ratio of 97 to 3 (Table S1, ESI†), in good agreement with the experimental result of 99% to <5% conversion.

#### Analysis of the diastereoselectivity

**Fig. 8:**
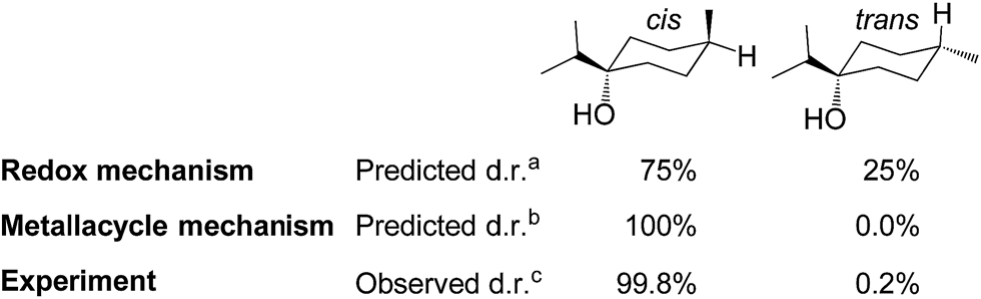
Computed diastereoselectivity of **C1**-catalysed hydrogenation of terpinen-4-ol, assuming a redox or metallacycle mechanism, and comparison to experiment (^a^Table S2, ESI, ^b^Table S3, ESI,† ^c^data from ref. 2f).

The product formed from hydrogenation of terpinen-4-ol can exist as two different diastereomers, which, respectively, have the ^i^Pr and methyl substituents *cis* or *trans* to each other (Fig. 8). In our computations, the *trans*-diastereomer is 1.9 kcal mol^–1^ lower in energy than the *cis*-diastereomer, yet in experiment, the *cis*-diastereomer is predominantly formed, with a very high diastereoselectivity of 99.8 : 0.2 (Fig. 1). This indicates that the hydroxyl group may have a directing effect that favours formation of the *cis*-isomer. We have here evaluated if the two discussed mechanisms, the metallacycle mechanism and the alternative redox pathway, are able to reproduce the experimentally observed diastereoselectivity.

In the redox mechanism as depicted in Fig. 3, the diastereoselectivity is determined in the insertion step (**TS_2-3R_**), where the OH substituent is oriented towards cobalt, leading to the *cis*-diastereomer. For formation of the *trans*-diastereomer, we have analysed four pathways (see ESI, Fig. S8–S11†), and find that the lowest pathway proceeds *via* β-hydrogen elimination (Fig. S10, ESI†). The overall barriers for formation of the *cis* and *trans*-diastereomers *via* a redox pathway differ by 0.6 kcal mol^–1^, which corresponds to a predicted d.r. of 75(*cis*) : 25(*trans*) (Fig. 8, Table S2 ESI†).

In the metallacycle mechanism, the diastereoselectivity is determined in the hydride transfer step **TS_1-2M_** (Fig. 5). Due to the strong coordination of the alkoxide oxygen, hydride addition inevitably has to lead to formation of only the *cis*-product (Fig. 8), in excellent agreement with the experimentally observed high d.r. of 99.8(*cis*) : 0.2(*trans*). Formation of the *trans*-diastereomer would require cleavage of the cobalt–alkoxide bond, which energetically is very costly (Table S3 ESI†). We propose that in the experimental reaction, the *cis*-diastereomer is formed *via* the energetically preferred metallacycle mechanism, whereas the very small amount of observed *trans*-diastereomer (0.2%, Fig. 1) must be formed *via* other mechanisms. Our computational results provide a rationale for the high diastereoselectivity of the cobalt complex **C1**. Interestingly, the Crabtree iridium catalyst provides the same diastereoselectivity with terpinen-4-ol.^16^ However, the mechanistic details of said system are not known so far.

## Experimental results

Hydrogenation of deuterium-labelled terpinen-4-ol (C_10_H_8_-OD) afforded 1,2-H_2_ alkane with no deuterium incorporation into the alkene double bond (Fig. S12, ESI†), consistent with the proposed metallacycle mechanism, where proton transfer from a second substrate to the cobalt alkoxide turns over the catalytic cycle (**TS_5M_**, Fig. 5).

**Fig. 9:**
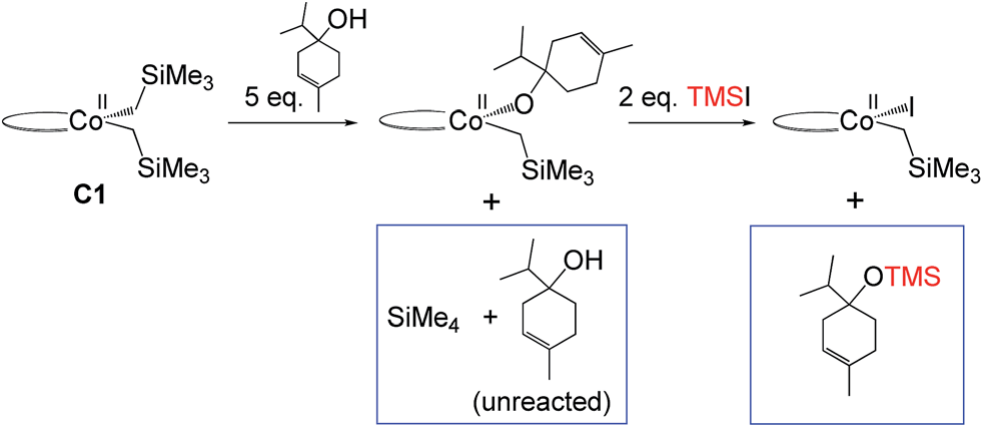
Indirect evidence for formation of an alkoxide intermediate. Components in boxes were identified *via* NMR (see ESI†).

Our attempts to obtain the metallacycle intermediate **2_M_** from different synthetic routes and to characterize it by X-ray crystallography were unsuccessful due to strong interference from the thermodynamically more accessible bis(ligand)cobalt species formed during isolation of cobalt complexes.^17^ However, indirect experimental evidence suggests that a reaction between the precatalyst dppeCo(CH_2_SiMe_3_)_2_ (**C1**) and terpinen-4-ol does occur (Fig. 9). Specifically, adding five equivalents of terpinen-4-ol to **C1** in benzene-d_6_ at room temperature resulted in protonolysis of the cobalt alkyl. The volatile component of the reaction was distilled and analysed by ^1^H-NMR after 12 hours, and the protonolysis product SiMe_4_ was observed.^18^ Integration suggests approximately half of the alkyl groups in **C1** reacted with terpinen-4-ol (Fig. S13, ESI†). This experimental observation supports the formation of a mono-alkoxide intermediate, in agreement with the mono-alkoxide species expected during the metallacycle mechanism (**1_M_**, Fig. 5). Further treatment of the non-volatile component (containing the assumed mono-alkoxy complex) with TMSI resulted in formation of TMS-terpinen-4-ol (Fig. 9), providing additional support for formation of an alkoxide species.

Treating precatalyst **C1** with H_2_ in the absence of substrate unveiled a catalyst deactivation pathway, which involves formation of a catalytically inactive (dppe)_2_Co species (Fig. S14, ESI†). This observation suggests an essential role of the substrate in maintaining the catalyst in an active form.

## Conclusions

We have here reported a new mechanism for bis(phosphine)-cobalt-catalysed hydrogenation of hydroxylated alkenes, proceeding through an energetically low-lying metallacycle intermediate (**2_M_**, Fig. 5). In the computational analysis, the metallacycle mechanism correctly predicts the preference for hydroxylated alkenes (Fig. 7) and the high diastereomeric ratio observed in hydrogenation of terpinen-4-ol (Fig. 8). The mechanism is further supported by experimental investigations providing indirect evidence of a cobalt-terpinen-4-ol alkoxide intermediate (Co-OR, Fig. 9).

Our computational analysis further shows that a previously proposed redox mechanism^14^ may be valid for non-hydroxylated substrates, but is unable to explain the activating effect and diastereoselectivity of hydroxylated alkenes. As also reported for iron-catalysed hydrogenation of carbonyl substrates,^19^ we have here shown that known substrate selectivities provide a straightforward tool for testing the validity of proposed mechanisms, and we suggest to always asses these important parameters in computational studies of reaction pathways.

## Conflicts of interest

There are no conflicts to declare.

## Supplementary Material

Supplementary information

Supplementary information
