# Supplementary material for: Cobalt-catalysed alkene hydrogenation: a metallacycle can explain the hydroxyl activating effect and the diastereoselectivity
Source: Chem Sci. 2018 May 4;9(22):4977–82. doi: 10.1039/c8sc01315b (PMC5989653; doi:10.1039/c8sc01315b)
Supplement: Supplementary file 2 [file SC-009-C8SC01315B-s002.pdf]

Electronic Supplementary Material (ESI) for Chemical Science.
This journal is © The Royal Society of Chemistry 2018

SUPPORTING INFORMATION

Cobalt-Catalysed Alkene Hydrogenation: A Metallacycle Can Explain the Hydroxyl Activating Effect and the Diastereoselectivity

Glenn R. Morello[a], Hongyu Zhong[b], Paul J. Chirik[b], and Kathrin H. Hopmann[a]

[a]Hylleraas Centre for Quantum Molecular Sciences, Department of Chemistry, University of Tromsø – The Arctic University of Norway, N-9037 Tromsø, Norway.

[b]Department of Chemistry, Princeton University, New Jersey 08544, United States.

CONTENT:

1. COMPUTAIONAL DETAILS

2. COBALT DIHYDRIDE VS COBALT DIHYDROGEN INTERMEDIATE

3. COMPUTATION OF ALTERNATE MECHANISMS FOR TERPINEN-4-OL

4. COMPUTATIONAL ANALYSIS OF PRECATALYST ACTIVATION

5. COMPUTED FORMATION OF 1_R FROM 1_M

6. MECHANISTIC PATHWAYS FOR α-METHYLSTYRENE

7. MECHANISTIC ANALYISIS OF FORMATION OF DIASTEREOMERS FROM TERPINEN-4-OL

8. COMPUTED SELECTIVITIES

9. EXPERIMENTAL DETAILS

10. NMR SPECTRA

1. COMPUTATIONAL DETAILS

Full molecular models of the catalyst **C1** and the terpinen-4-ol substrate were employed (Figure 1 in the main text). All calculations were performed with the Gaussian 09 package Rev. D.01[i] and the B3LYP hybrid density functional.[ii] Dispersion corrections were added to all calculations through use of the Grimme empirical dispersion correction GD3.[iii] To obtain a better description of the reaction in solution, the IEFPCM solvation model[iv] was used for all calculations, including geometry optimizations. Toluene was chosen as the solvent to match experimental parameters. All calculations were performed assuming a doublet electronic state for the cobalt complex. For geometry optimizations, the basis set 6-311G(d,p) (BS1) was employed.[v] Single point electronic energies were computed with the basis set 6-311++G(2df,2pd) (BS2).

All optimised geometries and transition states (TS) were confirmed by the presences of zero and one imaginary frequency, respectively, in the calculated Hessian. Gibbs free energies were determined at 298.15 K and 1 atm utilizing unscaled vibrational frequencies. The final free energies were determined as $\Delta G_{final} = \Delta G_{raw} - \Delta EBS1 + \Delta EBS2$.

2. COBALT DIHYDRIDE COMPARED TO COBALT DIHYDROGEN INTERMEDIATE

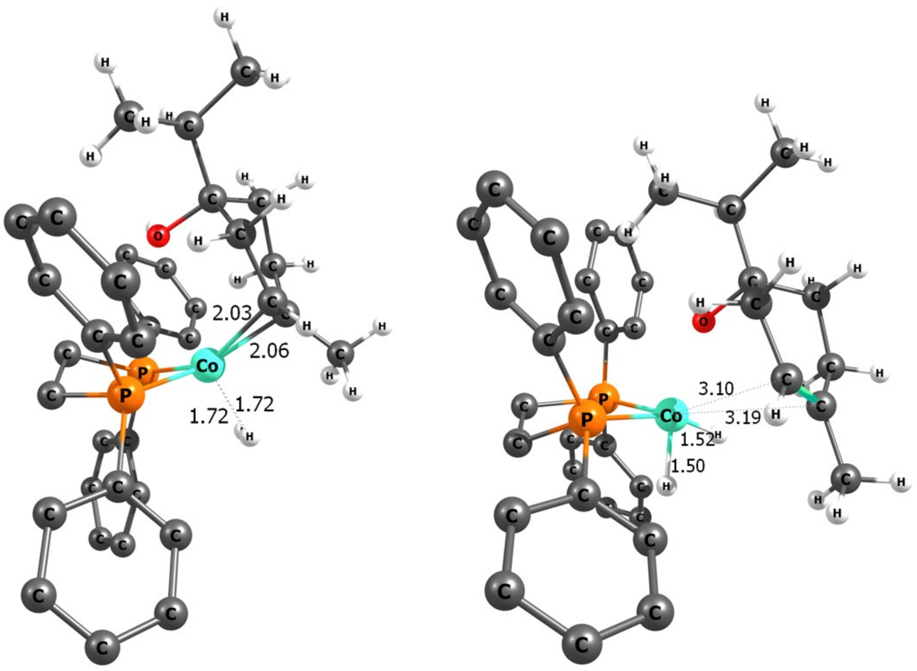

Relative Energy = **0.0** **+2.6**

Figure S1. Geometric and energetic differences between the Co^0 species, $\mathbf{2_R}$, (*left*) and the Co^{II} version of the same intermediate (*right*), where the H_2 atom has been split into a dihydride. Hydrogen atoms of (dppe) ligand removed for clarity. Bond lengths reported in Å, energies given in kcal/mol.

3. ALTERNATE MECHANISMS

3.1. ALTERNATE REDOX MECHANISM 1: HYDRIDE TRANSFER TO THE NON-METHYLATED CARBON

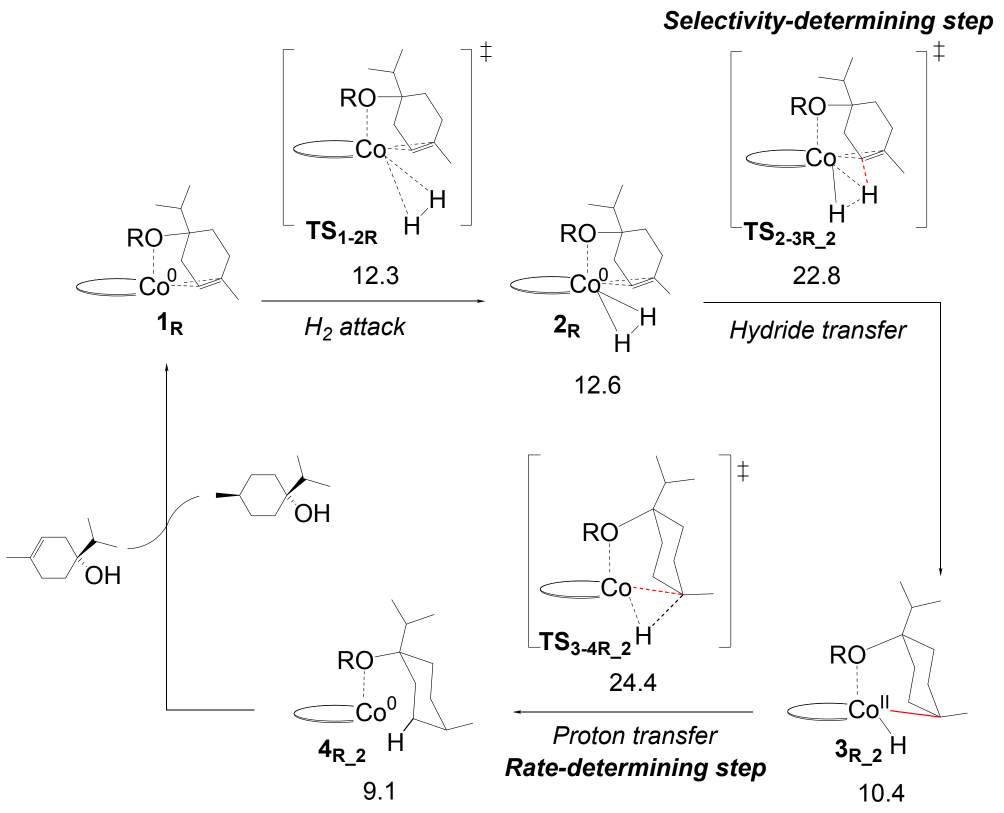

Figure S2. Computed free energies (kcal/mol) for hydrogenation of terpinen-4-ol to the cis-product via a mechanism in which the hydride transfer occurs to the non-substituted sp^2 carbon atom of the substrate (**TS**$_{2\text{-}3R\_2}$) resulting in cobalt coordination to the methylated carbon atom. This is followed by proton transfer to the methylated carbon (**TS**$_{4\text{-}5R\_2}$). The overall barrier for this mechanism (24.4 kcal/mol) exceeds the redox pathway described in Figure 3 of the main text (barrier of 23.6 kcal/mol).

3.2. ALTERNATE REDOX MECHANISM 2: BETA-HYDRIDE ELIMINATION

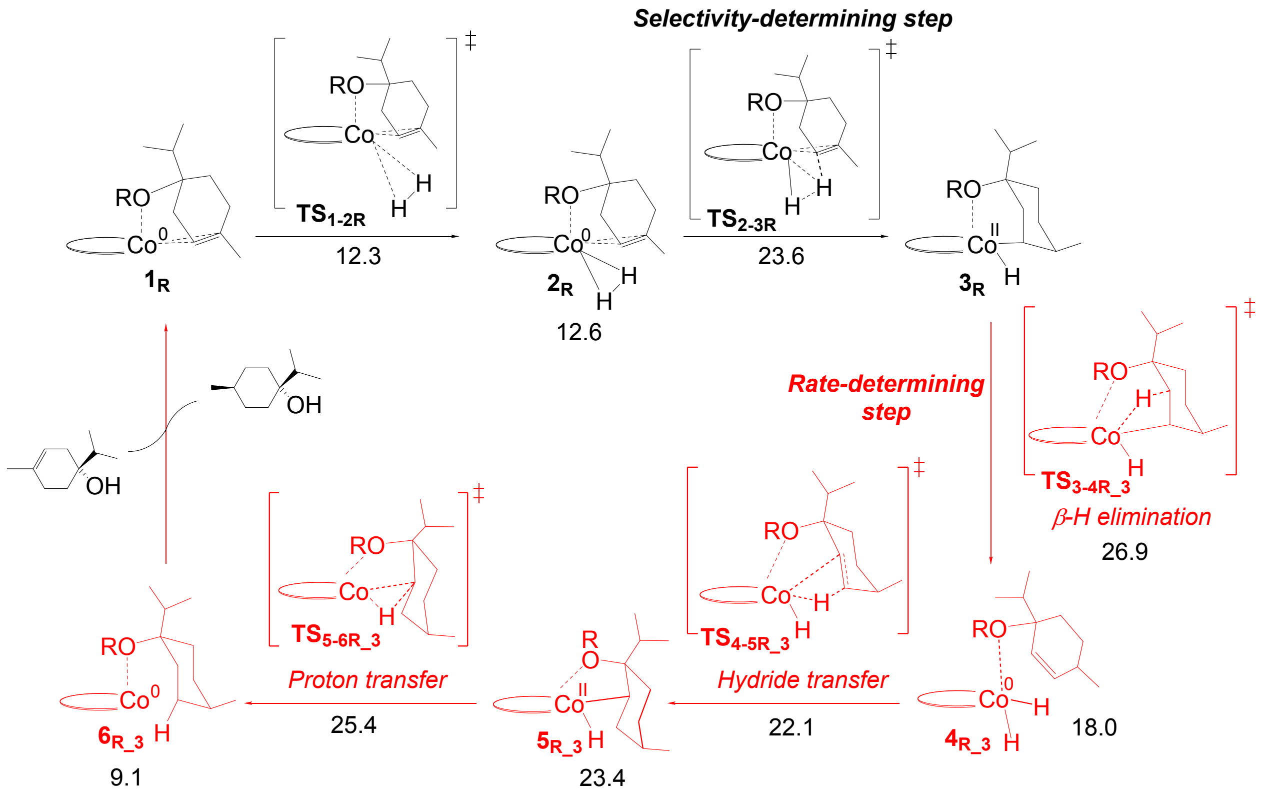

Figure S3. Computed free energies (kcal/mol) for hydrogenation of terpinen-4-ol to the cis-product via a mechanism in which hydride transfer occurs to the methyl-substituted sp^2 carbon atom of the substrate (**TS**$_{2\text{-}3R}$) followed by β-hydride elimination from the carbon atom adjacent to the OR substituted carbon, **TS**$_{3\text{-}4R\_3}$. The overall barrier for this mechanism (26.9 kcal/mol) exceeds the redox pathway described in Fig. 3 of the main text (barrier 23.6 kcal/mol). For optimised coordinates, see XYZ file.

3.3 ALTERNATE NON-REDOX MECHANISM 3: σ-BOND METATHESIS

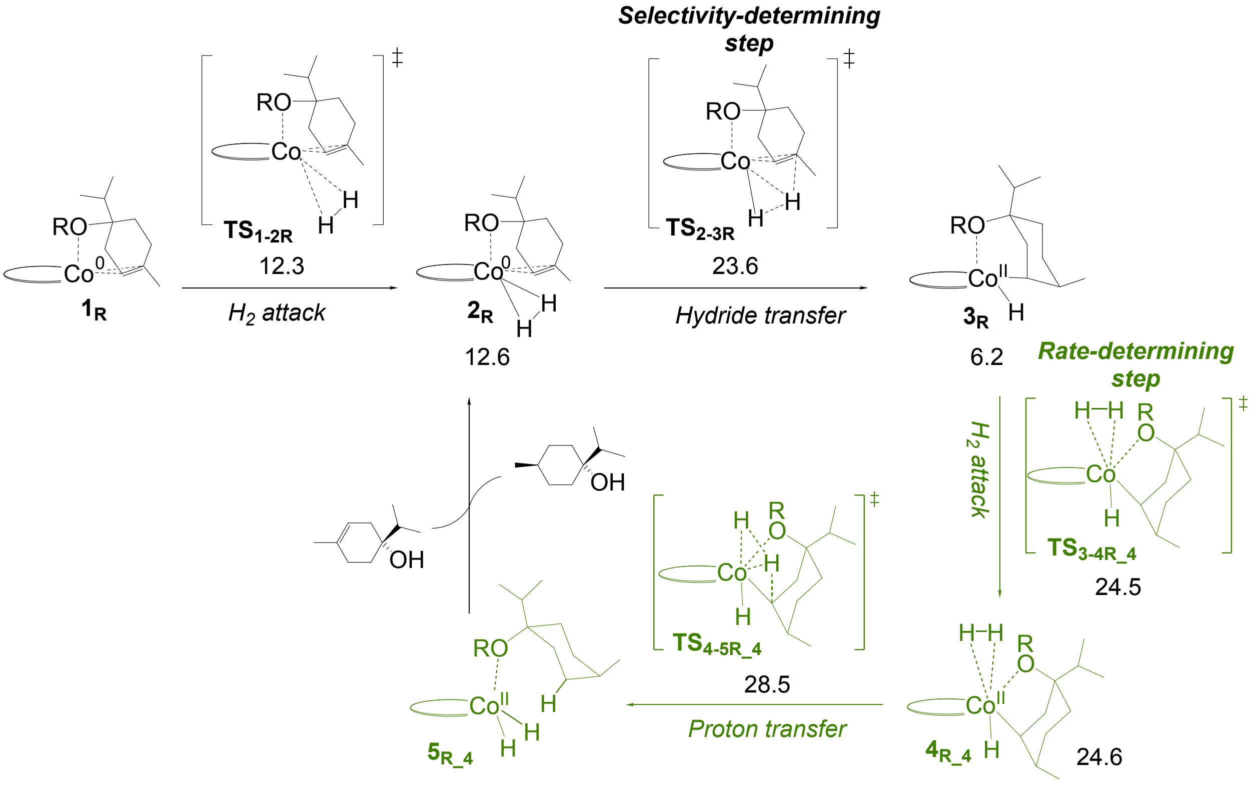

Figure S4. Computed free energies (kcal/mol) for hydrogenation of terpinen-4-ol to the *cis*-product via a non-redox σ-bond metathesis mechanism, in which the hydride transfer occurs to the methyl-substituted sp[2] carbon atom of the substrate (**TS$_{2\text{-}3R}$**), followed by H_2 coordination (**TS$_{3\text{-}4R_4}$**) and proton transfer to the substrate (**TS$_{4\text{-}5R_4}$**). The overall barrier for this mechanism (28.5 kcal/mol) exceeds the redox pathway described in Figure 3 of the main text (barrier of 23.6 kcal/mol). For optimised coordinates, see XYZ file.

4. GENERATION OF THE ACTIVE CATALYST

We predict that generation of the active species **1_M** occurs through σ-bond metathesis from the precatalyst **C1** (Fig. S5). The starting trimethylsilyl (TMS) complex can undergo ligand substitutions by either splitting H_2 or by activating the O-H bond of a substrate molecule, each liberating one TMS group. H_2 splitting (barrier of 29.3 kcal/mol) followed by splitting of an OH group (barrier 7.7 kcal/mol) leads to formation of **1_M**, with a relative energy of -32.3 kcal/mol. Formation of the dihydride complex has a higher barrier for the second step (12.0 kcal/mol) and results in a species of higher energy (-23.6 kcal/mol). This indicates that in presence of OH-bearing substrates, formation of **1_M** is preferred over formation of the previously proposed dihydride species. Formation of a bis-alkoxide species (-28.9 kcal/mol) has a higher barrier (32.5 kcal/mol) than initial H_2 splitting (29.3 kcal/mol).

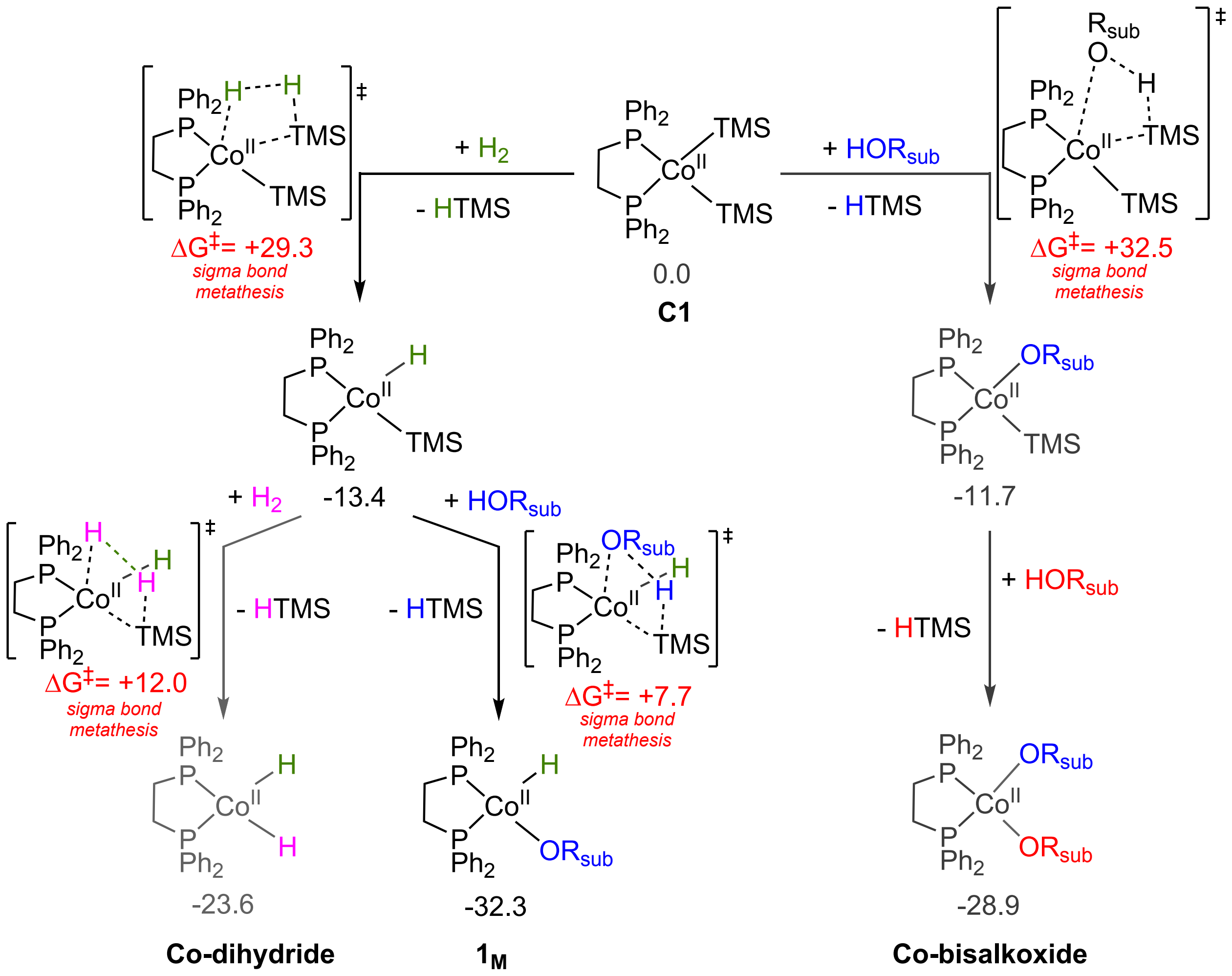

Figure S5. Energetic flow chart (kcal/mol) comparing transformation of the precatalyst **C1** to either a dihydride, the mono-alkoxide **1_M** or a bis-alkoxide.

5. REDUCTIVE ELIMINATION OF ALCOHOL SUBSTRATE TO FORM COBALT(0) SPECIES 1_R

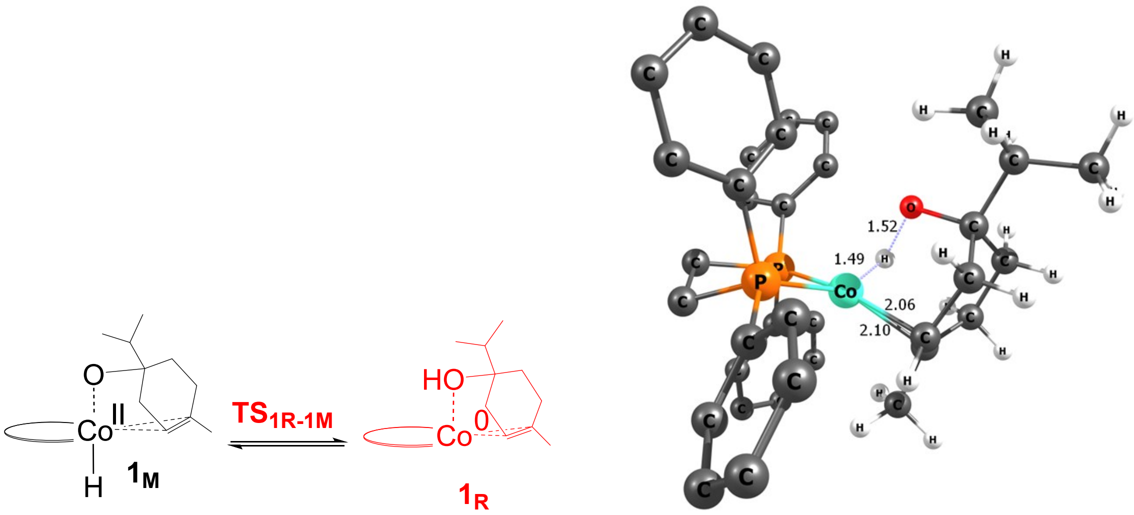

Figure S6. Left: Reductive elimination of the oxygen-coordinated substrate in complex $\mathbf{1_M}$ to form the Co(0) species $\mathbf{1_R}$. Right: Optimised geometry of the transition state $\mathbf{TS_{1R\text{-}1M}}$, with a computed activation energy ($\Delta G^{\ddagger}$) of +35.5 kcal/mol relative to $\mathbf{2_M}$ (the lowest point of the metallacycle mechanism, Figure 4, main text). Hydrogen atoms of the pincer ligand removed for clarity. Bond angles reported in degrees, bonds in Å.

6. MECHANISTIC PATHWAYS FOR α-METHYLSTYRENE

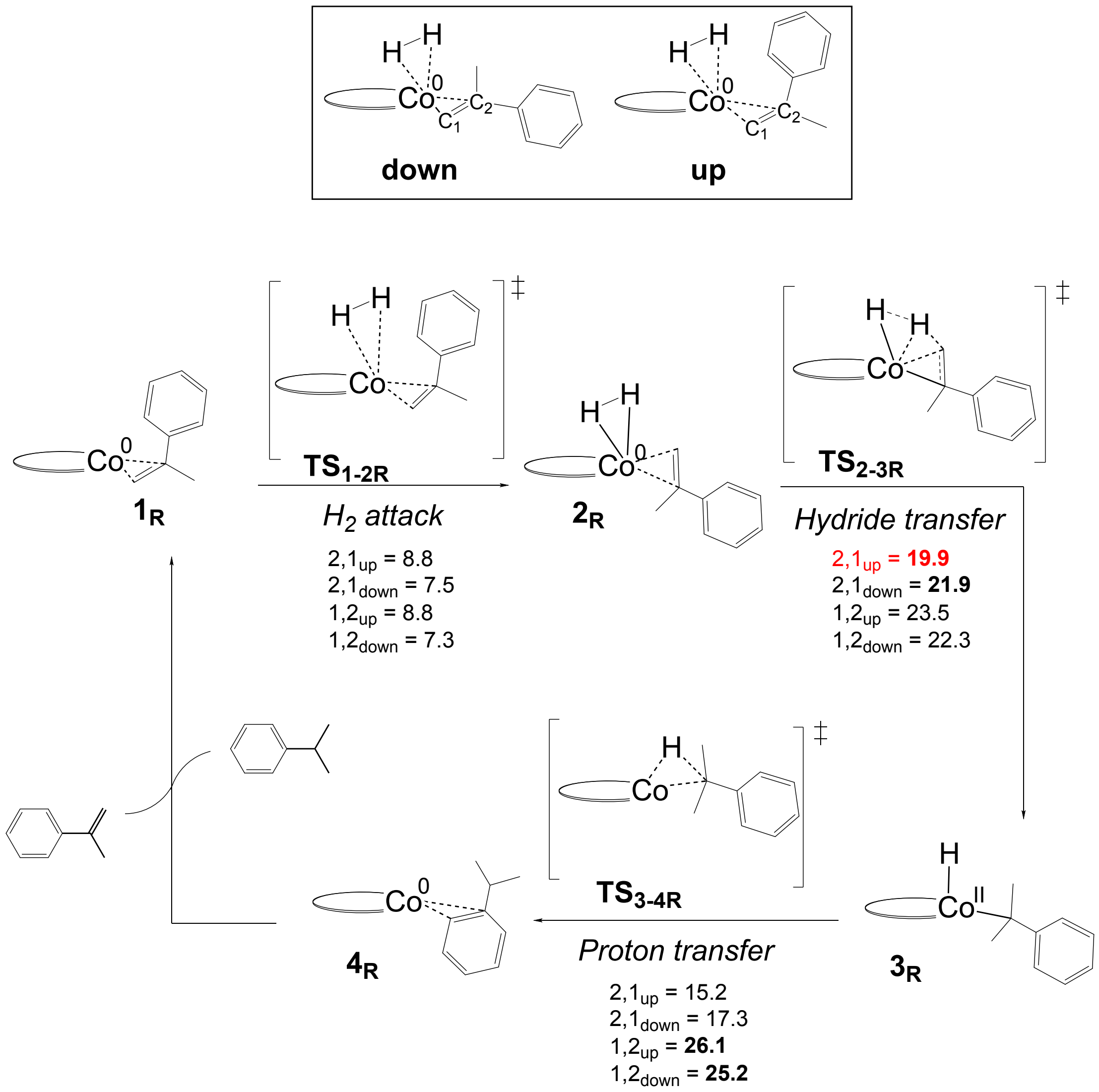

Figure S7. Computed free energies (kcal/mol) for hydrogenation of α-methylstyrene via 2,1- or 1,2-insertion, with two different substrate orientations (up or down).

7. MECHANISTIC ANALYISIS OF FORMATION OF DIASTEREOMERS FROM TERPINEN-4-OL

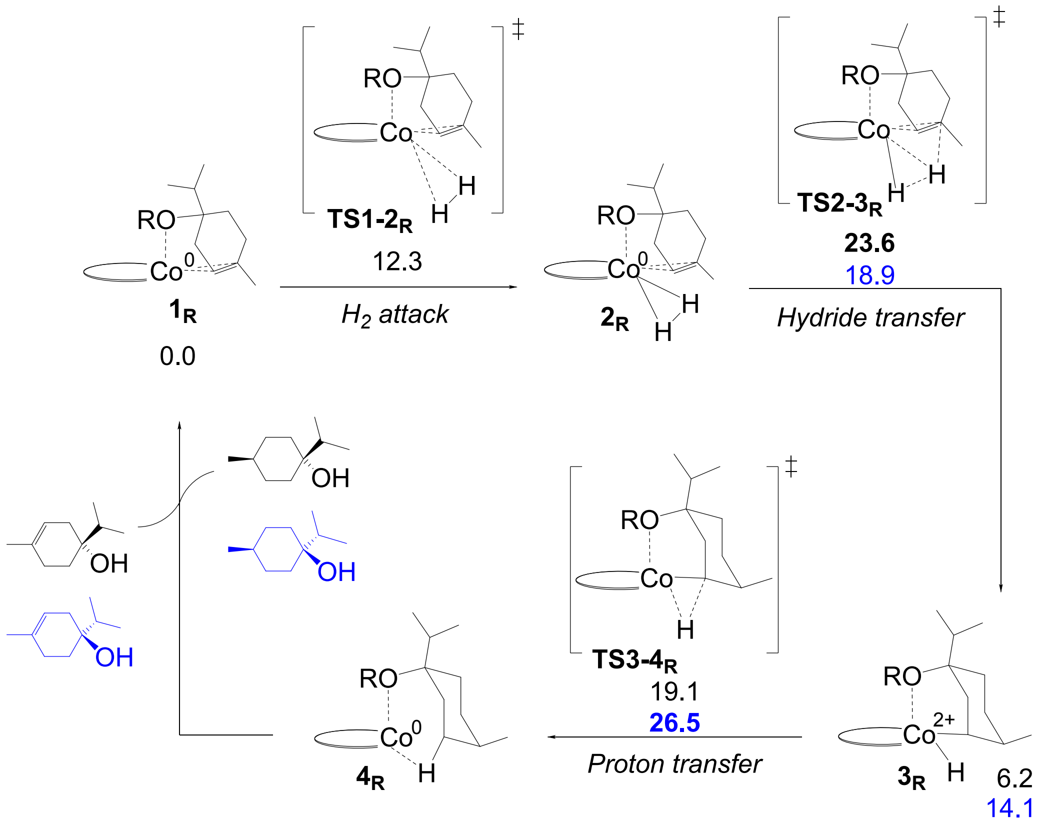

Figure S8. Comparison of computed free energies (kcal/mol) for hydrogenation of terpinen-4-ol to the *cis*- or *trans*-product, respectively, via a mechanism in which the hydride transfer occurs to **the methyl-substituted sp² carbon** atom of the substrate. Here black represents the *cis*-product, while blue represents the *trans*-product. Note that this is the most favorable pathway computed for formation of the *cis*-product (barrier 23.6 kcal/mol, **TS$_{2-3R}$**). The schematic drawings reflect the *cis*-geometries. For optimised coordinates, see XYZ file.

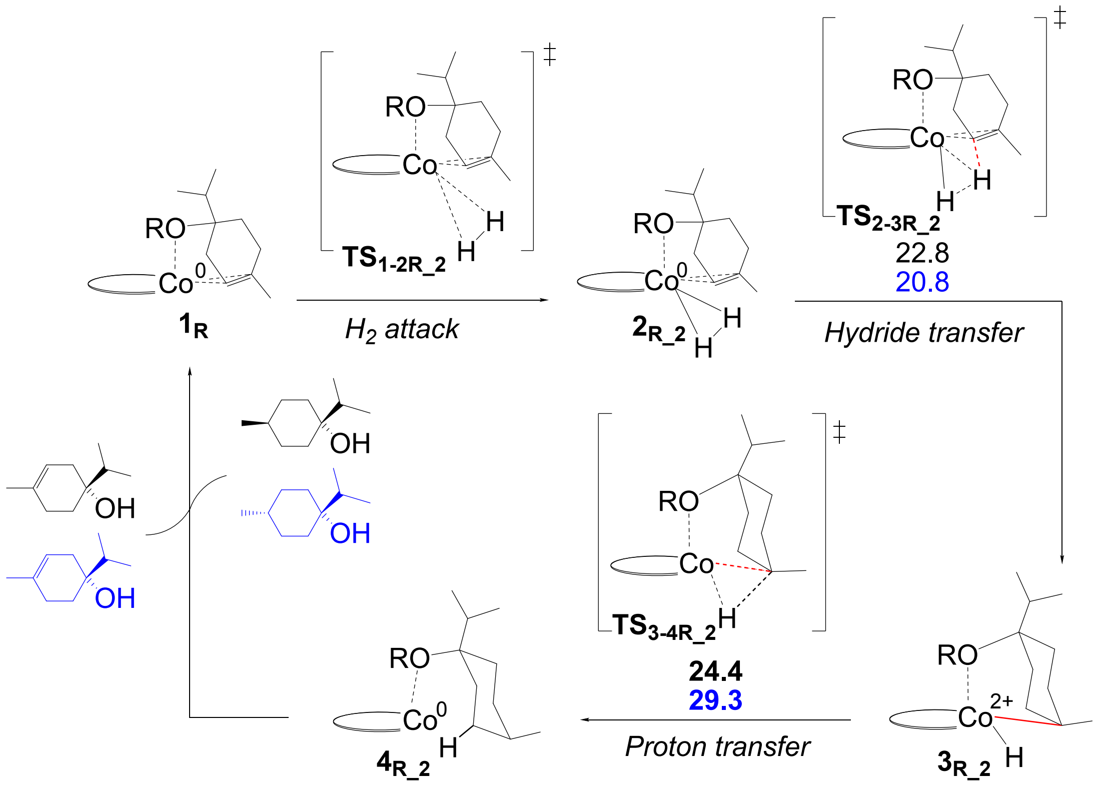

Figure S9. Comparison of computed free energies (kcal/mol) for hydrogenation of terpinen-4-ol to the *cis*- or *trans*-product, respectively, via a mechanism in which the hydride transfer occurs to the **non-substituted sp² carbon atom** of the substrate (**TS$_{2-3R_2}$**). This is followed by proton transfer to the methylated carbon (**TS$_{4-5R_2}$**). Here black represents the *cis*-product, while blue represents the *trans* product. The schematic drawings reflect the *cis*-geometries. For optimised coordinates, see XYZ file.

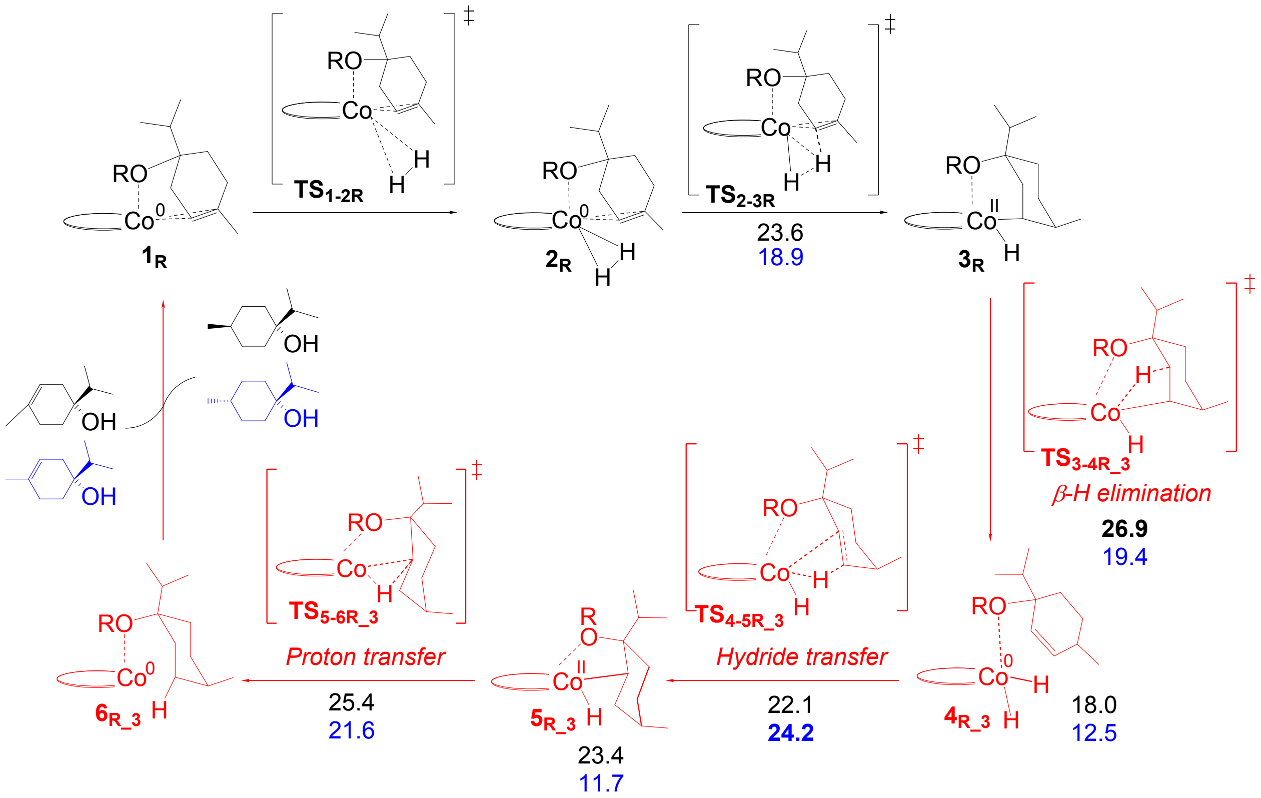

Figure S10. Comparison of computed free energies (kcal/mol) for hydrogenation of terpinen-4-ol to the *cis*- or *trans*-product, respectively, via a mechanism in which the hydride transfer occurs to the methyl-substituted sp^2 carbon atom of the substrate ($TS_{2\text{-}3R}$) **followed by β-H elimination from the carbon atom adjacent to the OR substituted carbon, $TS_{3\text{-}4R\_3}$**. Black represents the *cis*-product, blue the *trans*-product. Note that this is the most favorable pathway computed for formation of the *trans*-product (barrier 24.2 kcal/mol relative to 1_R). The drawings reflect the *cis*-geometries. For optimised coordinates, see XYZ file.

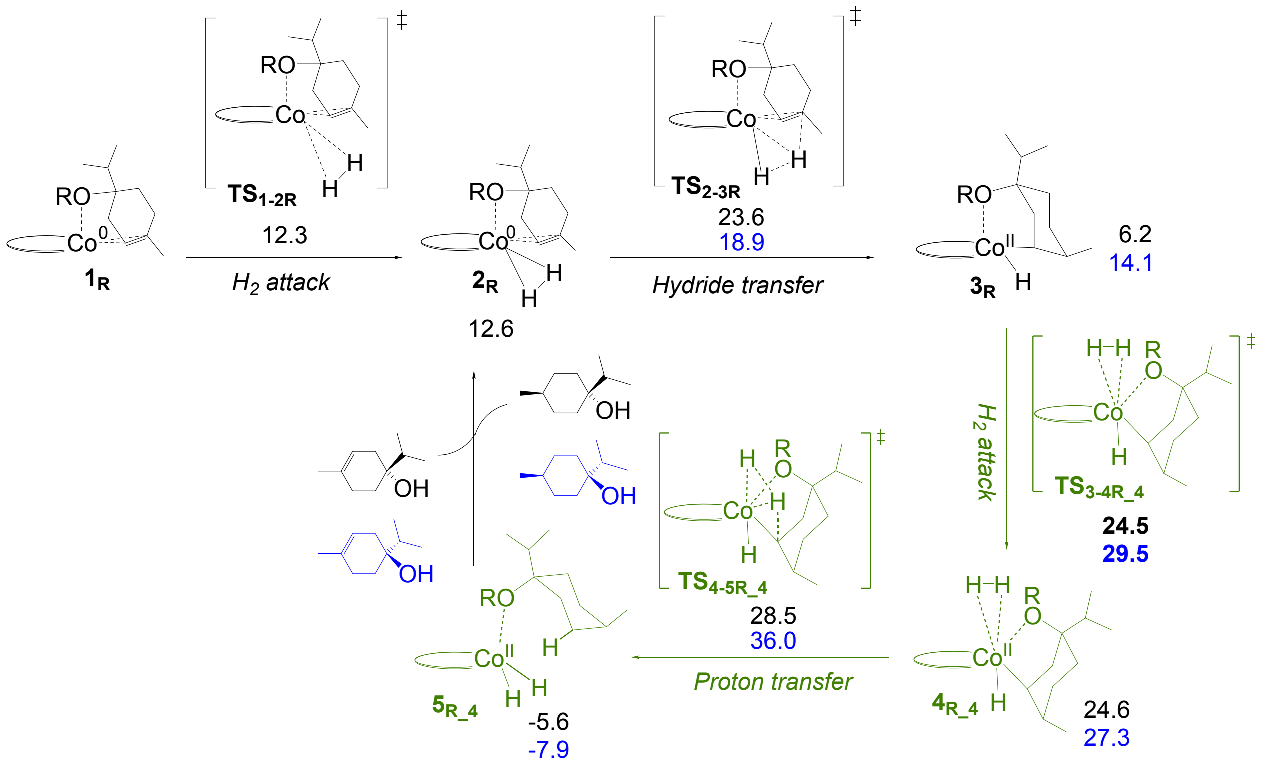

Figure S11. Comparison of computed free energies (kcal/mol) for hydrogenation of terpinen-4-ol to the *cis*- or *trans*-product, respectively, **via a non-redox σ-bond metathesis mechanism** in which the hydride transfer occurs to the methyl-substituted sp^2 carbon atom of the substrate ($TS_{2\text{-}3R}$), followed by H_2 coordination ($TS_{3\text{-}4R\_4}$) and proton transfer to the substrate ($TS_{4\text{-}5R\_4}$). Black represents the *cis*-product, blue represents the *trans*-product. The drawings reflect the *cis*-geometries. For optimised coordinates, see XYZ file.

8. COMPUTED SELECTIVITIES

The selectivities were computed on basis of barrier differences between different pathways (ΔΔG≠) employing equation 1: $[A]/[B] = \exp(\Delta\Delta G^{‡}_{B-A}/RT)$

Table S1. Substrate selectivity for terpinen-4-ol versus its methoxy derivative

	ΔG‡ for formation of *cis*-product from terpinen-4-ol via metallacycle pathway (Fig.4 main text)	ΔG‡ for formation of *cis*-product from OMe-derivative of terpinen-4-ol via redox pathway (Fig.4 main text)	ΔΔG‡ [$\Delta G^{‡}_{OMe}$- $\Delta G^{‡}_{OH}$]	Product ratio (eq 1)
Formation of experimentally observed *cis*-product from terpinen-4-ol or OMe derivative	21.3 kcal/mol	23.3 kcal/mol	2 kcal/mol	97(OH): 3(OMe)

Table S2. Diastereoselectivity for redox pathway

	ΔG‡ for formation of cis-product from terpinen-4-ol via redox pathway (Fig.4 main text)	ΔG‡ for formation of *trans*-product from terpinen-4-ol via redox β-H pathway (Fig. S10)	ΔΔG‡ [$\Delta G^{‡}_{trans}$- $\Delta G^{‡}_{cis}$]	Product ratio (eq 1)
Formation of *cis*- or *trans*-product from terpinen-4-ol	23.6 kcal/mol (relative to 1_R)	24.2 kcal/mol (relative to 1_R)	0.6 kcal/mol	75(*cis*): 25(*trans*)

Table S3. Diastereoselectivity for metallacycle pathway

	ΔG‡ for formation of *cis*-product from terpinen-4-ol via metallacycle pathway (Fig.4 main text)	ΔG‡ for formation of *trans*-product from terpinen-4-ol via metallacycle pathway	ΔΔG‡ [$\Delta G^{‡}_{trans}$- $\Delta G^{‡}_{cis}$]	Product ratio (eq 1)
Formation of *cis*-product or trans product from terpinen-4-ol	21.3 kcal/mol	49.6 kcal/mol	28.3 kcal/mol	100(*cis*): 0(*trans*)

9. EXPERIMENTAL DETAILS

General Information: All air- and moisture-sensitive manipulations were carried out using standard high vacuum line or in an M. Braun glovebox under N_2 atmosphere. The M. Braun glovebox was equipped with a cold well designed for freezing samples at liquid nitrogen temperature. Solvents for air- and moisture-sensitive manipulations were dried and deoxygenated using literature procedures.[vi] Deuterated solvents for NMR spectroscopy were distilled from sodium metal under an atmosphere of argon prior to use. Anhydrous cobalt dichloride was purchased from Sigma-Aldrich and used as received. 1,2-Bis(diphenylphosphino)ethane ligand was purchased from Strem Chemicals and used as received. Terpinen-4-ol was purchased from Sigma-Aldrich and purified by dynamic vacuum distillation followed by passage through a pad of calcium hydride and celite and storage under an inert atmosphere under room temperature.

^{1}H-NMR and ^{13}C-NMR were recorded on a Bruker NanoBay-300(300 MHz ^{1}H), or Bruker Avance-500(500 MHz ^{1}H and 125 MHz ^{13}C). ^{1}H chemical shifts are reported relative to $SiMe_4$ using ^{1}H chemical shifts of the residual protio solvent as a secondary standard. ^{13}C chemical shifts are reported relative to $SiMe_4$ using chemical shifts of the solvent as a secondary standard.

Gas chromatography for the alkane products was performed on a Shimadzu GC-2010 gas chromatograph. GC analyses were performed using a Restek 15 m x 0.25 mm RTX-5 5% diphenyl/95% dimethyl polysiloxane column with a film thickness of 0.25 µm.

Single crystals suitable for X-ray diffraction were coated with polyisobutylene oil in a glovebox, transferred to a nylon loop and then quickly transferred to the goniometer head of a Bruker D8 APEX3 Venture diffractometer equipped with a molybdenum X-ray tube (λ = 0.71073 Å) and a Cu X-ray tube (λ = 1.54178 Å). Preliminary data revealed the crystal system. The data collection strategy was optimised for completeness and redundancy using the Bruker COSMO software suite. The space group was identified, and the data were processed using the Bruker SAINT+ program and corrected for absorption using SADABS. The structures were solved using direct methods (SHELXS) completed by subsequent Fourier synthesis and refined by full-matrix least-squares procedures.

Experimental Procedures: The catalyst dppeCo($CH_2SiMe)_3$ (**C1**) was synthesised using a previously reported method.[vii] Catalytic hydrogenation reactions were carried out in thick-walled glass vessels under 1 or 4 atm H_2 using the previously reported procedures.[vii]

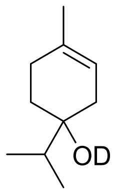

Synthesis of deuterium-labeled terpinen-4-ol: In a nitrogen-filled glovebox, a 20 mL scintillation vial was charged with 1.00 g (6.48 mmol) of terpinen-4-ol, 10 mL of THF and a stir bar. The solution was cooled at 77 K. ^{n}BuLi solution (7.13 mmol, 2.8 mL, 2.5 M in hexane) was diluted using 10 mL hexanes and cooled at 77 K. Into the thawing solution of terpinen-4-ol, the ^{n}BuLi solution was added dropwise. The mixture was stirred at room temperature for 1 hour, and 1 mL of CD_3OD (99.8% atom %D) was added dropwise to the mixture. The resulting solution was stirred at r.t. for 15 minutes, and the volatiles were removed *en vacuo*. The residue was reconstituted in toluene, filtered through celite followed by passage through a thin pad of alumina. The volatiles were removed *en vacuo* to afford (0.94 g, 93% yield) of deuterium-labeled terpinen-4-ol ($C_{10}H_8$-OD). ^{1}H-NMR(500 MHz, $CDCl_3$): δ 5.21 (m, 1H, H_b), δ 2.03 (m, 2H), δ 1.75 (m, 2H), δ 1.58 (s, 3H, H_a), δ 1.52 (m, 2H), δ 1.34 (m, 1H), δ 1.09 (s, 0.04H, residual OH), δ 0.93 (d, 3H, H_g), δ 0.88 (d, 3H, $H_{g'}$). 96% deuterium incorporation (96:4 $C_{10}H_8$-OD : $C_{10}H_8$-OH) was identified based on ^{1}H-NMR integration.

Labeling experiments supports 1,2-H_2 addition across C=C bond without incorporation of alcohol proton consistent with proposed mechanism: Hydrogenation of deuterium-labeled terpinen-4-ol was carried out using standard procedure.[vii]

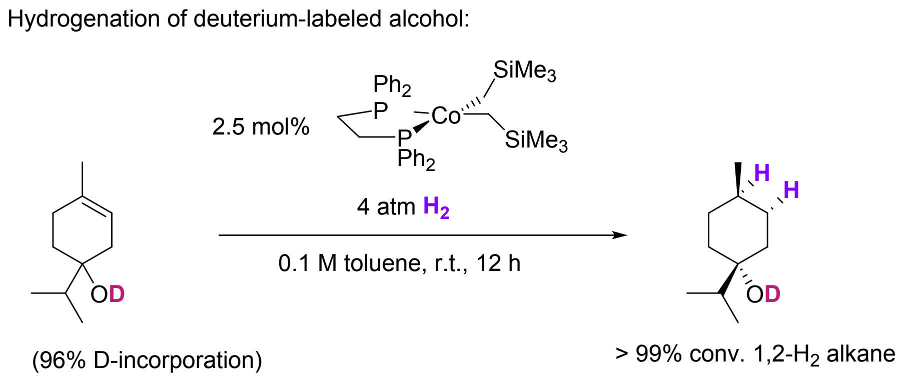

Figure S12. Hydrogenation of deuterium-labeled terpinen-4-ol. ^{1}H-NMR integration suggests 1,2-H_2 alkane product. No observable deuterium-attached carbons were found in ^{13}C-NMR.

Evidence for protonolysis of cobalt dialkyl by terpinen-4-ol and formation of cobalt alkoxide: In a nitrogen-filled glovebox, a 20 mL scintillation vial was charged with 0.015 g of dppeCo(CH_2SiMe_3)$_2$, 0.5 mL of benzene-d_6. The resulting solution was transferred into a J-Young tube and cooled at 77 K. Into another 20 mL scintillation vial was charged with 0.018 g of terpinen-4-ol and 0.5 mL of benzene -d_6. The resulting solution was cooled at 77 K, and upon thawing, transferred into the J-Young tube. The tube was quickly sealed, brought outside the box, and let stand at room temperature for 12 hours. Vacuum distillation of the volatiles of the reaction content (benzene-d_6, $SiMe_4$, unreacted terpinen-4-ol) under high vacuum into another J-Young tube was performed, and ^{1}H-NMR of the volatiles identifies both $SiMe_4$ product and unreacted excess terpinen-4-ol. The non-volatile components were brought into the glovebox, transferred into a 20 mL-scintillation vial in THF. The volatiles were removed, affording 0.016 g of residue. Into the residue was added 5 mL THF and 0.095 g (2.0 equiv, assuming mono-alkoxy product based on ^{1}H-NMR integration of volatiles) of TMSI. The mixture was stirred at r.t. for 12 h, and the volatiles (THF, unreacted TMSI) was removed *en vacuo*. The residue (dppeCo(CH_2SiMe_3)$_x$(I)$_n$, TMS-terpinen-4-ol) was dissolved in benzene-d_6 into a J-Young tube, and the contents were distilled on high vacuum line into another J-Young tube. ^{13}C-NMR of the volatiles confirmed the identity of TMS-terpinen-4-ol.

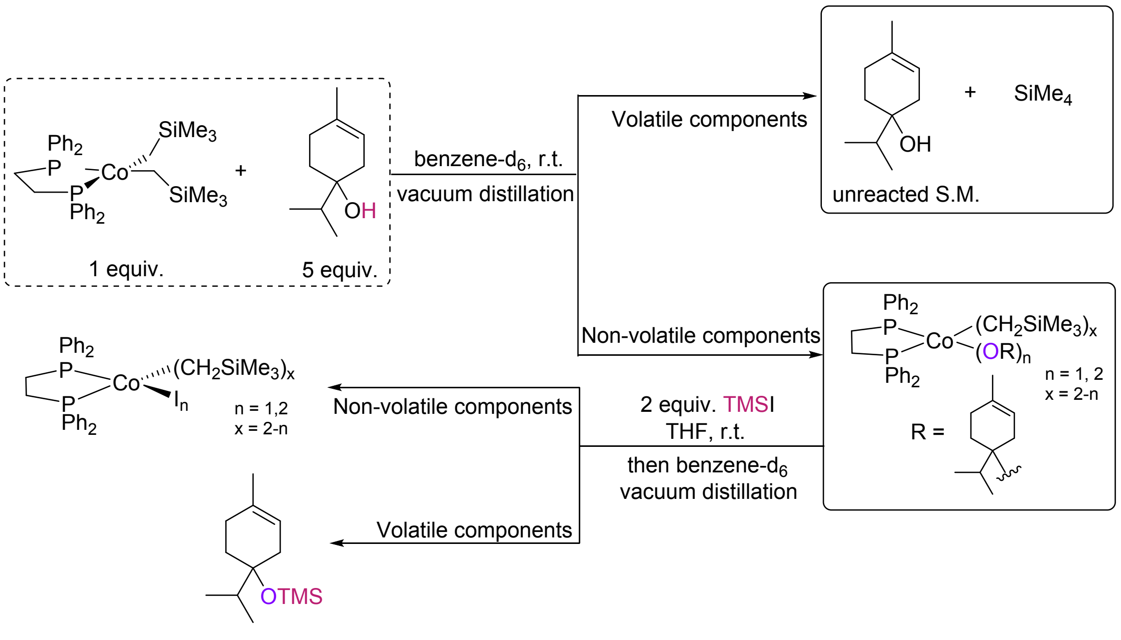

Figure S13. Reaction between dppeCo(CH_2SiMe_3)$_2$ and substrate occurs at room temperature to generate $SiMe_4$ and a proposed cobalt alkoxide species

Catalyst deactivation occurs from reacting with H_2 in the absence of substrate: In a nitrogen-filled glovebox, a thick-walled glass vessel was charged with 0.100 g of dppeCoCH$_2$SiMe$_3$, 5 mL Et$_2$O, a stir bar and sealed. The vessel was bought outside the box and attached to a high vacuum line. The contents of the vessel were frozen in liquid nitrogen, and the head space of N_2 was removed. Then 4 atm H_2 was added to the vessel and sealed. The mixture was stirred for 15 minutes at room temperature and the contents were frozen in liquid nitrogen again. The headspace of H_2 was removed and the vessel was brought back into the glovebox. The mixture was concentrated *en vacuo*, affording a red solid in 0.086 g yield and was sampled for ^{1}H-NMR analysis. The solid was tested for catalytic activity, and hydrogenation of terpinen-4-ol using the sample solid at 2.5 mol% loading afforded no desired alkane product. Crystallization from a saturated pentane solution of the red solid at -35 °C overnight afforded crystals suitable for x-ray diffraction.

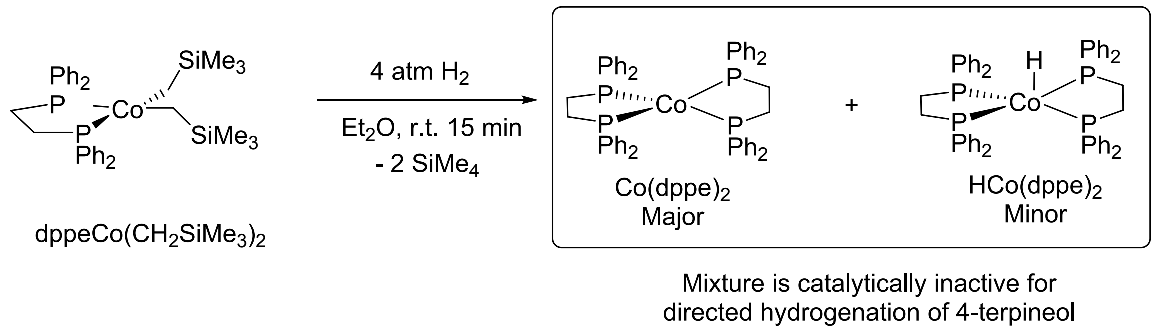

Figure S14. Treating dppeCo(dialkyl) with H_2 in the absence of substrate leads to rapid formation of catalytically inactive bis(ligand)-cobalt species.

The mixture of (dppe)$_2$Co and HCo(dppe)$_2$ was isolated and observed by ^{1}H-NMR. (dppe)$_2$Co was also crystallographically characterised (Figure S15). The complex (dppe)$_2$Co was first reported by Sacco.[viii] The complex HCo(dppe)$_2$ was first reported by Sacco[ix] and was synthesised from addition of NaBH$_4$ to dppeCoBr$_2$. The complex HCo(dppe)$_2$ was crystallographically characterised by DuBois.[x] (dppe)$_2$Co was reported to reduce H_2 at atmospheric pressure in benzene solution to give the Co(I) monohydride HCo(dppe)$_2$.[viii]

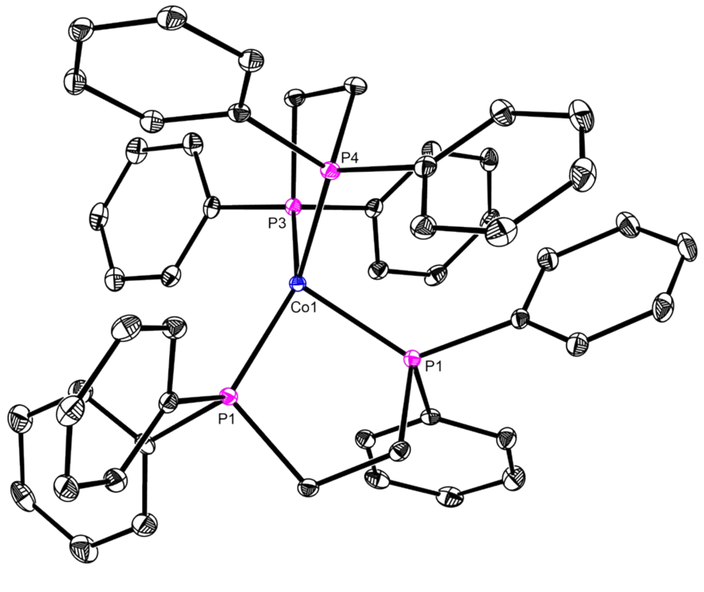

Figure S15. X-ray structure of (dppe)$_2$Co at 30% probability ellipsoids with H atoms omitted for clarity

10. NMR SPECTRA

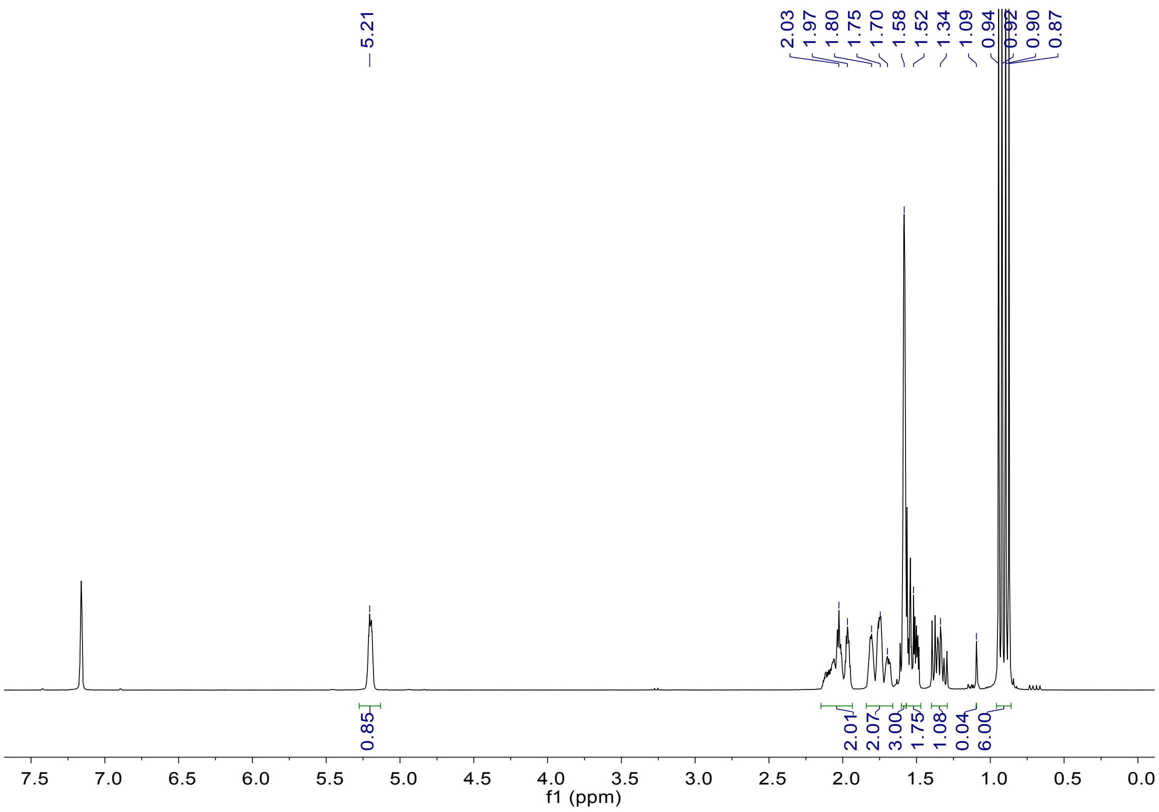

Figure S16. ^{1}H-NMR spectrum of deuterium-labeled terpinen-4-ol.

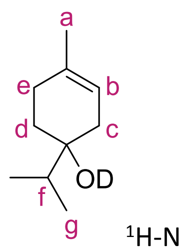

^{1}H-NMR(500 MHz, $CDCl_3$): δ 5.21 (m, 1H, H_b), δ 2.03 (m, 2H), δ 1.75 (m, 2H), δ 1.58 (s, 3H, H_a), δ 1.52 (m, 2H), δ 1.34 (m, 1H), δ 1.09 (s, 0.04H, residual OH), δ 0.93 (d, 3H, H_g), δ 0.88 (d, 3H, $H_{g'}$). 96:4 $C_{10}H_8$-OD : $C_{10}H_8$-OH based on ^{1}H-NMR integration.

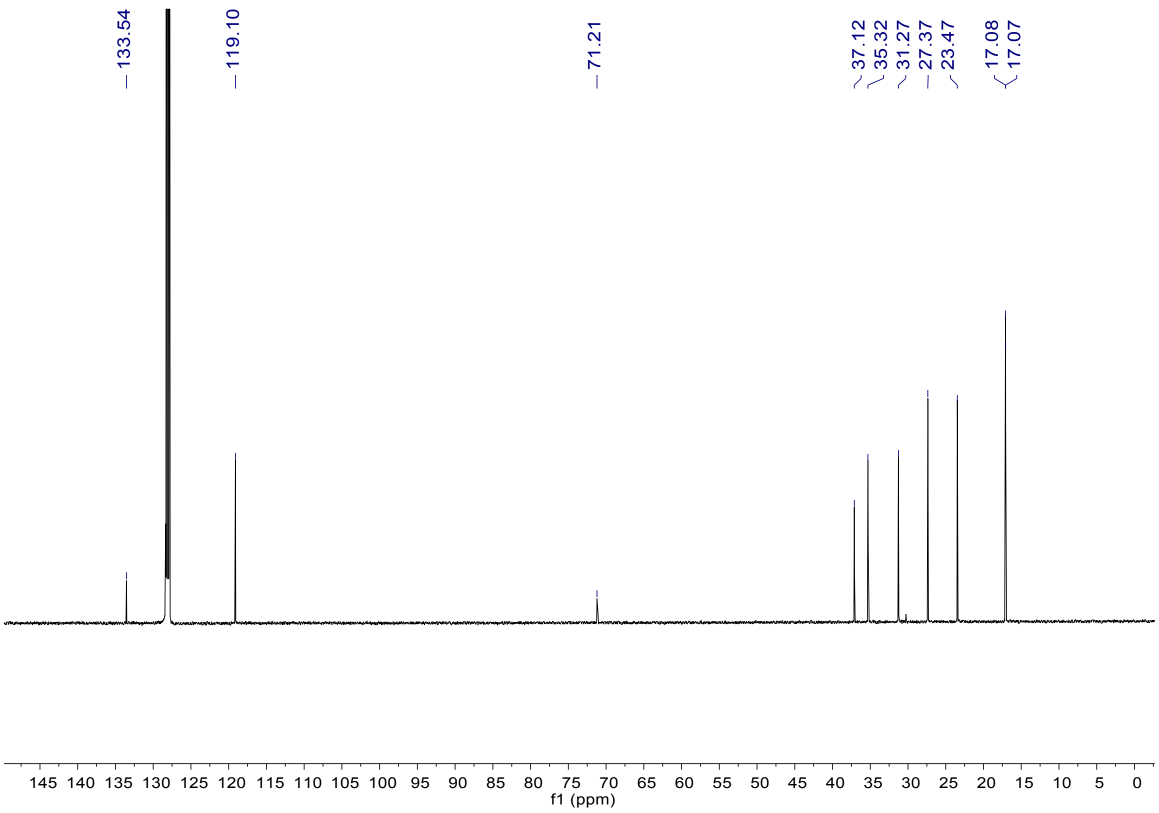

Figure S17. ^{13}C-NMR spectrum of deuterium-labeled terpinen-4-ol.

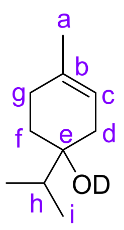

^{13}C-NMR(125 Hz, $CDCl_3$): δ 133.5 (b), δ 119.1 (c), δ 71.2 (e), δ 37.1, δ 35.3, δ 31.3, δ 23.5, δ17.1 (i, i’).

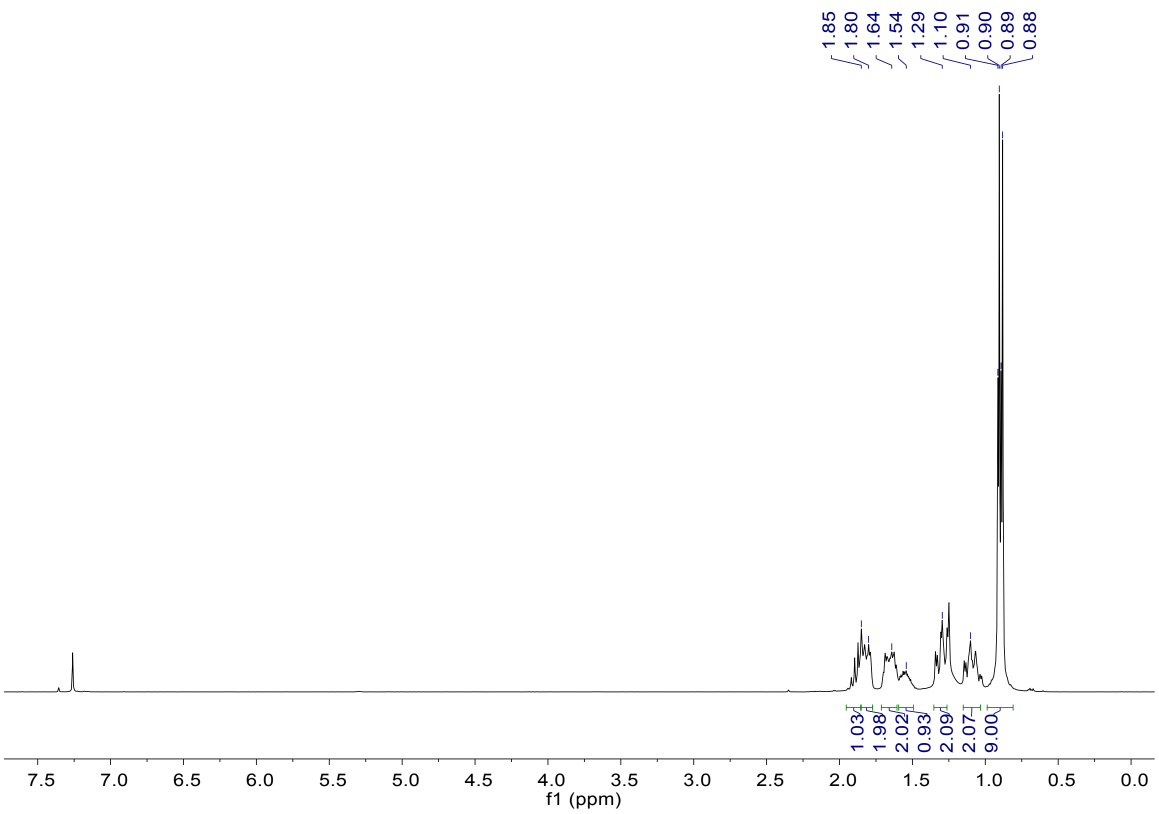

Figure S18. ^{1}H-NMR spectrum of hydrogenation product of deuterium-labeled terpinen-4-ol.

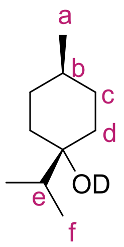

^{1}H-NMR (500 MHz, $CDCl_3$): δ 1.85 (hept, 1H, H_e, overlapping), δ 1.80 (m, 2H), δ 1.64 (m, 2H), δ 1.54 (m, 1H, H_b), δ 1.29 (m, 2H), δ 1.10 (m ,2H), δ 0.90 (d, 3H, H_a, overlapping), δ 0.89 (d, 6H, H_f, overlapping). Proton integration matches 1,2-H_2 alkane with no deuterium incorporation.

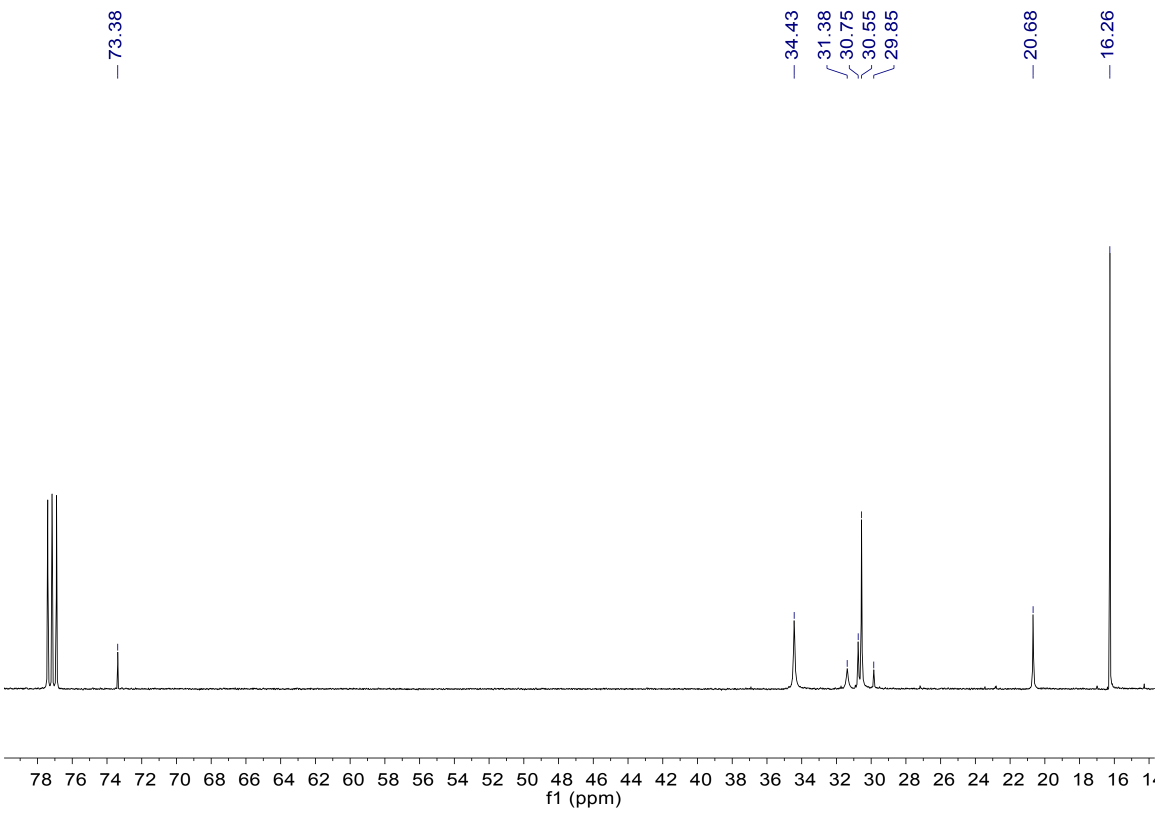

Figure S19. ^{13}C-NMR spectrum of hydrogenation product of deuterium-labeled terpinen-4-ol.

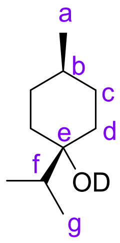

^{13}C-NMR (500 MHz, $CDCl_3$): δ 73.4 (C_e), δ 34.4 (C_b), (δ 31.4 grease), δ 30.8 (C_f), δ 30.6 (C_c), δ 29.9 (C_d), δ 20.7 (C_a), δ 16.3 (C_g).

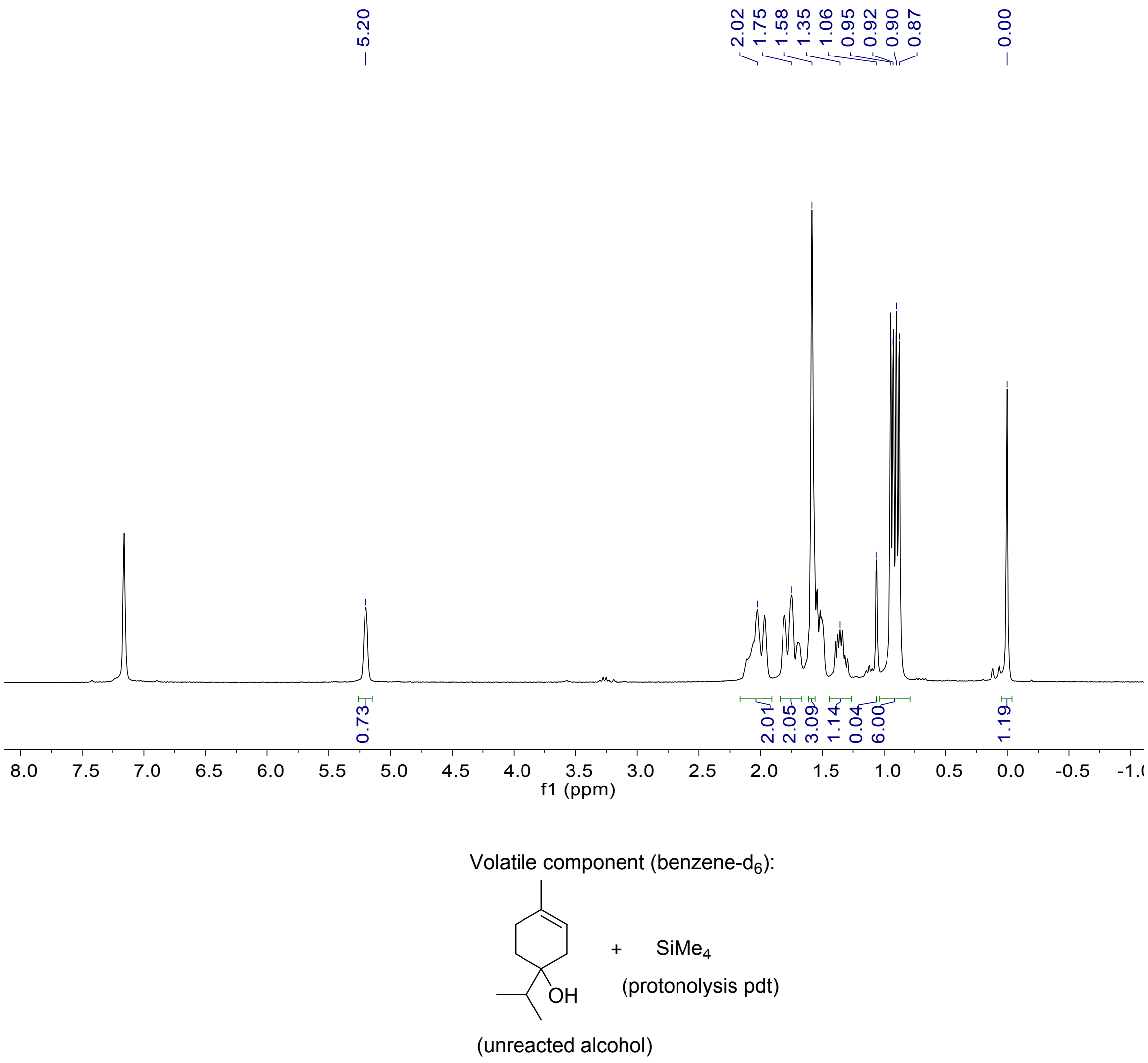

Figure S20. ^{1}H-NMR evidence for reaction between dppeCo(CH_2SiMe_3)$_2$ and terpinen-4-ol and generation of $SiMe_4$. 49% of -CH_2SiMe_3 group in dppeCo(CH_2SiMe_3)$_2$ reacted with terpinen-4-ol based on ^{1}H-NMR integration of generated $SiMe_4$ and residual terpinen-4-ol.

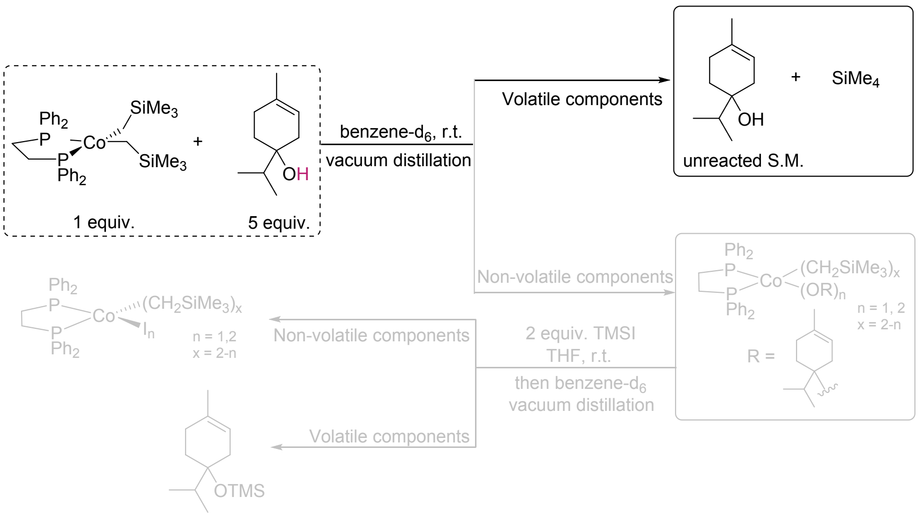

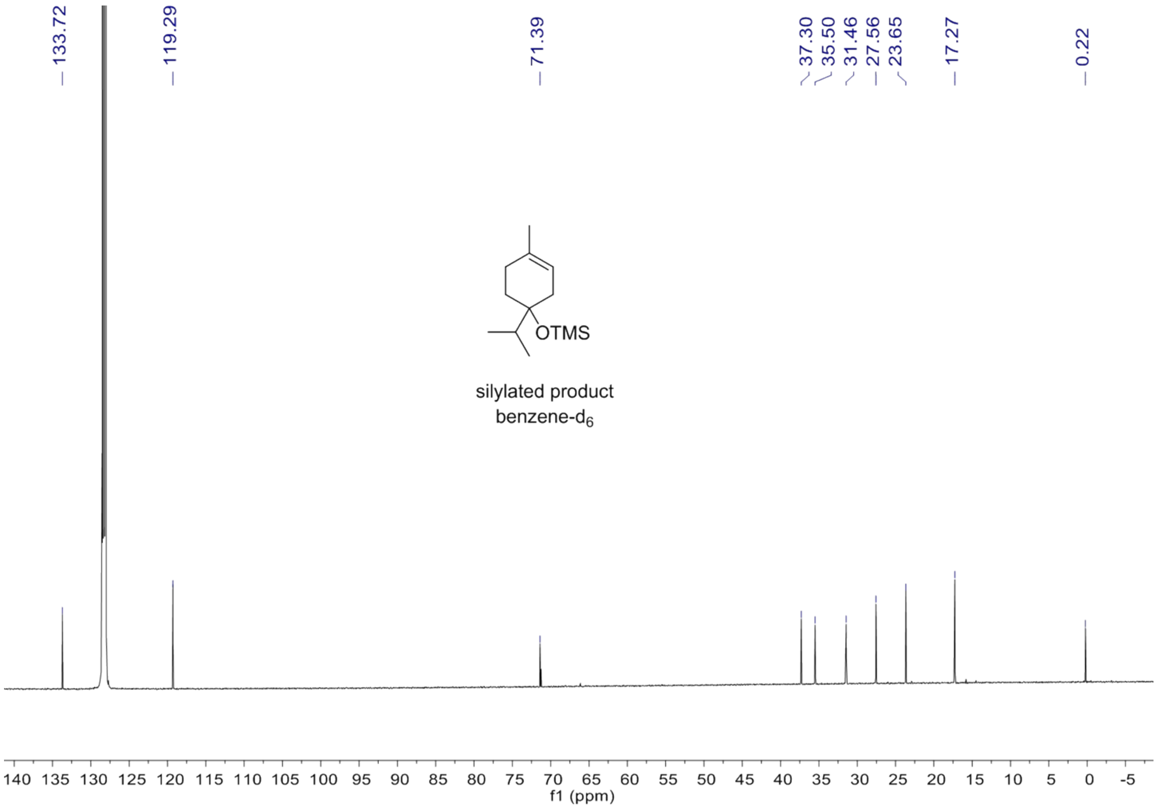

Figure S21. ^{13}C-NMR evidence for formation of dppeCo$(OR)_n(CH_2SiMe_3)_x$ and its reaction with TMSI generating TMS-terpinen-4-ol

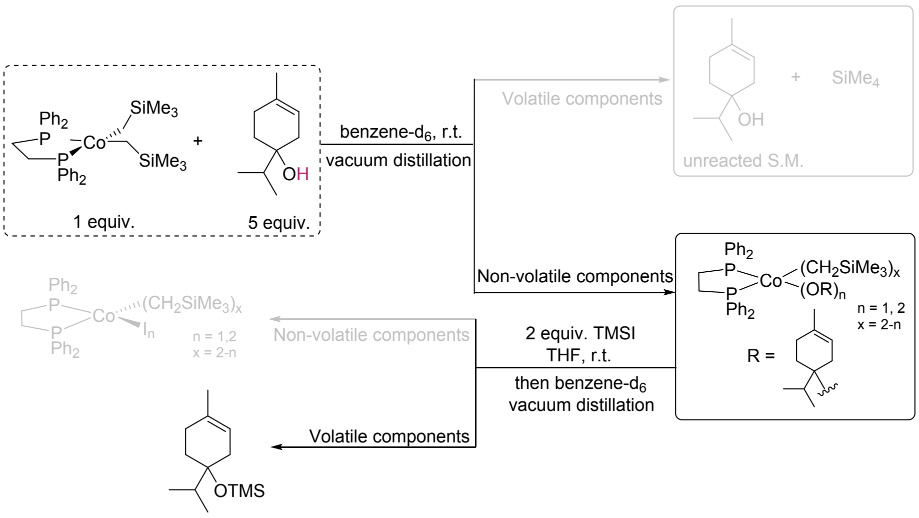

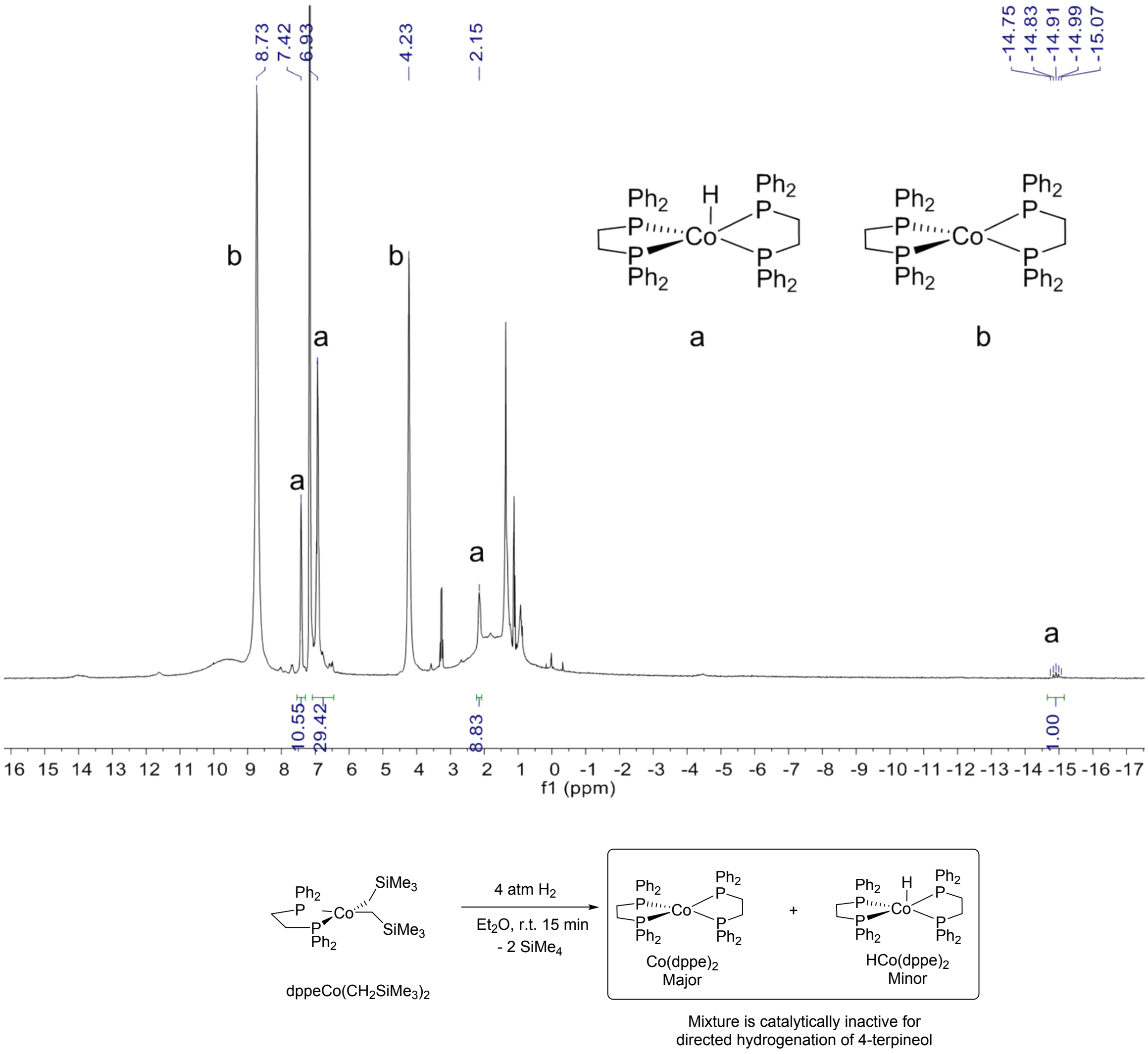

Figure S22. ^{1}H-NMR for a mixture of $(dppe)_2Co$ and $H(dppe)_2Co$ from catalyst deactivation

References:

[i] Gaussian 09, Revision D.01, Frisch, M. J.; Trucks, G. W.; Schlegel, H. B.; Scuseria, G. E.; Robb, M. A.; Cheeseman, J. R.; Scalmani, G.; Barone, V.; Mennucci, B.; Petersson, G. A.; Nakatsuji, H.; Caricato, M.; Li, X.; Hratchian, H. P.; Izmaylov, A. F.; Bloino, J.; Zheng, G.; Sonnenberg, J. L.; Hada, M.; Ehara, M.; Toyota, K.; Fukuda, R.; Hasegawa, J.; Ishida, M.; Nakajima, T.; Honda, Y.; Kitao, O.; Nakai, H.; Vreven, T.; Montgomery, J. A., Jr.; Peralta, J. E.; Ogliaro, F.; Bearpark, M.; Heyd, J. J.; Brothers, E.; Kudin, K. N.; Staroverov, V. N.; Kobayashi, R.; Normand, J.; Raghavachari, K.; Rendell, A.; Burant, J. C.; Iyengar, S. S.; Tomasi, J.; Cossi, M.; Rega, N.; Millam, J. M.; Klene, M.; Knox, J. E.; Cross, J. B.; Bakken, V.; Adamo, C.; Jaramillo, J.; Gomperts, R.; Stratmann, R. E.; Yazyev, O.; Austin, A. J.; Cammi, R.; Pomelli, C.; Ochterski, J. W.; Martin, R. L.; Morokuma, K.; Zakrzewski, V. G.; Voth, G. A.; Salvador, P.; Dannenberg, J. J.; Dapprich, S.; Daniels, A. D.; Farkas, Ö.; Foresman, J. B.; Ortiz, J. V.; Cioslowski, J.; Fox, D. J. Gaussian, Inc., Wallingford CT, 2009.

[ii] (a) Becke, A. D. Phys. Rev. A **1988**, *38*, 3098-3100, (b) Lee, C.; Yang, W.; Parr, R.G., Phys. Rev. B **1988**, *37*, 785-789.

[iii] Grimme, S.; Antony, J.; Ehrlich, S.; Krieg, H. *J. Chem. Phys*. **2010**, ***132*,** 154104.

[iv] a) Tomasi, J.; Mennucci, B.; Cammi, R. *Chem. Rev.* **2005**, *105*, 2999-3093, b) Tomasi, J.; Mennucci, B.; Cancès E. *J. Mol. Struct.: THEOCHEM* **1999**, *464*, 211-216, c) Cancès, E.; Mennucci, B.; Tomasi, J. *J. Chem. Phys.* **1997**, *107*, 3032-3041.

[v] Krishnan, R.; Binkley, J. S.; Seeger, R.; Pople, J. A. *J. Chem. Phys.* **1980,** *72*, 650-654.

[vi] Pangborn, A. B.; Giardello, M. A.; Grubbs, R. H.; Rosen, R. K.; Timmers, F. J. *Organometallics*, **1996**, *15*, 1518.

[vii] Friedfeld, M. R.; Margulieux, G. W.; Schaefer, B. A.; Chirik, P. J. *J. Am. Chem. Soc*., **2014**, *136*, 13178-13181.

[viii] Sacco, A; Rossi, M. *Chem. Commun*. (London) **1965**, *23*, 602-603.

[ix] Sacco, A.; Ugo, R. *J. Chem. Soc*. **1964**, 3274-3278.

[x] Ciancanelli, R.; Noll, B. C.; DuBois, D. L.; DuBois, M. R. *J. Am. Chem. Soc*. **2002**, *124*, 2984.
